# AMP-Activated Protein Kinase (AMPK) Regulates Energy Metabolism through Modulating Thermogenesis in Adipose Tissue

**DOI:** 10.3389/fphys.2018.00122

**Published:** 2018-02-21

**Authors:** Lingyan Wu, Lina Zhang, Bohan Li, Haowen Jiang, Yanan Duan, Zhifu Xie, Lin Shuai, Jia Li, Jingya Li

**Affiliations:** ^1^State Key Laboratory of Drug Research, Shanghai Institute of Materia Medica, Chinese Academy of Sciences, Shanghai, China; ^2^University of Chinese Academy of Sciences, Beijing, China; ^3^Shanghai Engineering Research Center of Molecular Therapeutics and New Drug Development, East China Normal University, Shanghai, China

**Keywords:** AMP-activated protein kinase, thermogenesis, energy metabolism, A-769662, white adipose browning, PGC-1α

## Abstract

Obesity occurs when excess energy accumulates in white adipose tissue (WAT), whereas brown adipose tissue (BAT), which is specialized in dissipating energy through thermogenesis, potently counteracts obesity. White adipocytes can be converted to thermogenic “brown-like” cells (beige cells; WAT browning) under various stimuli, such as cold exposure. AMP-activated protein kinase (AMPK) is a crucial energy sensor that regulates energy metabolism in multiple tissues. However, the role of AMPK in adipose tissue function, especially in the WAT browning process, is not fully understood. To illuminate the effect of adipocyte AMPK on energy metabolism, we generated Adiponectin-Cre-driven adipose tissue-specific AMPK α1/α2 KO mice (AKO). These AKO mice were cold intolerant and their inguinal WAT displayed impaired mitochondrial integrity and biogenesis, and reduced expression of thermogenic markers upon cold exposure. High-fat-diet (HFD)-fed AKO mice exhibited increased adiposity and exacerbated hepatic steatosis and fibrosis and impaired glucose tolerance and insulin sensitivity. Meanwhile, energy expenditure and oxygen consumption were markedly decreased in the AKO mice both in basal conditions and after stimulation with a β3-adrenergic receptor agonist, CL 316,243. In contrast, we found that in HFD-fed obese mouse model, chronic AMPK activation by A-769662 protected against obesity and related metabolic dysfunction. A-769662 alleviated HFD-induced glucose intolerance and reduced body weight gain and WAT expansion. Notably, A-769662 increased energy expenditure and cold tolerance in HFD-fed mice. A-769662 treatment also induced the browning process in the inguinal fat depot of HFD-fed mice. Likewise, A-769662 enhanced thermogenesis in differentiated inguinal stromal vascular fraction (SVF) cells via AMPK signaling pathway. In summary, a lack of adipocyte AMPKα induced thermogenic impairment and obesity in response to cold and nutrient-overload, respectively, whereas chronic AMPK activation by A-769662 promoted WAT browning in inguinal WAT and protected against HFD-induced obesity and related metabolic dysfunction. These findings reveal a vital role for adipocyte AMPK in regulating the browning process in inguinal WAT and in maintaining energy homeostasis, which suggests that the targeted activation of adipocyte AMPK may be a promising strategy for anti-obesity therapy.

## Introduction

Obesity, which has reached epidemic proportions globally (Finucane et al., 2011), is associated with disorders, including type II diabetes, cardiovascular disease, and some cancer. The main cause of obesity is a chronic imbalance between energy intake and energy expenditure. For many years, it was believed that two main types of adipose tissue exist in mammals: white adipose tissue (WAT) and brown adipose tissue (BAT). The primary function of WAT is to store excess energy as triglycerides, whereas BAT is responsible for dissipating chemical energy as heat through thermogenesis (Berry et al., 2013). BAT and skeletal muscle are two well-described thermogenic tissues that utilize different mechanisms to generate heat for maintaining normal core body temperature in cold environment (Rowland et al., 2015; Bal et al., 2017a). Skeletal muscle employs both shivering and nonshivering thermogenesis via various mechanisms including mitochondrial metabolism and futile ATP hydrolysis, while BAT-mediated nonshivering thermogenesis is extremely dependent on mitochondrial metabolism (Bal et al., 2012, 2017b). Recent studies have discovered another type of thermogenic adipose tissue called beige fat and have demonstrated that both classic brown adipocyte and beige adipocyte coexist in adult humans (van Marken Lichtenbelt et al., 2009; Wu et al., 2012; Rosen and Spiegelman, 2014). However, beige adipocytes possess some distinct characteristics over classic brown adipocytes. First, beige adipocytes have a relatively low basal level of uncoupling protein 1 (UCP1) but express a high level of UCP1 in response to cold and adrenergic stimulation. Second, beige adipocytes are not derived from the myf5^+^ lineage from which classic brown adipocytes originate. Third, beige adipocytes are readily induced by various environmental cues, such as chronic cold stimulation, exercise and agonists of pro-adipogenic or pro-thermogenic transcription factors that regulate beige adipogenesis or thermogenesis (Kajimura et al., 2015; Inagaki et al., 2016).

Evidence has shown that augmenting the activity or content of brown and beige fat is beneficial for boosting energy expenditure (Bartelt and Heeren, 2014). Therefore, therapeutics that target brown fat or remodel white fat into beige fat (referred to as WAT browning) for the treatment of obesity and its related metabolic diseases have gained clinical interest. In rodents, a number of tissues and cell types have been found to secrete factors that regulate WAT browning or thermogenesis in brown and beige adipose tissue, such as orexin (Sellayah et al., 2011), bone morphogenetic protein 7 (BMP7) (Tseng et al., 2008), catecholamine hormones such as norepinephrine (NE) secreted from sympathetic neurons (Collins, 2011), natriuretic peptides secreted from cardiac tissue (Bordicchia et al., 2012), fibroblast growth factor 21 (FGF21) secreted from the liver and BAT (Lee et al., 2014), PGC-1α-dependent myokine irisin (Boström et al., 2012), T4 secreted from the thyroid, and BMP8b and vascular endothelial growth factor (VEGF) secreted from BAT (Whittle et al., 2012; Bagchi et al., 2013). In addition to secreted factors, several small molecules have also been shown to induce WAT browning, such as a transient receptor potential cation channel subfamily V member 4 (TRPV4) antagonist that upregulates PGC-1α (Ye et al., 2012).

AMPK, a ubiquitously distributed serine/threonine protein kinase, regulates cellular energy homeostasis, acting as a central energy sensor and monitor by turning on catabolic pathways to generate ATP and turning off energy-consuming anabolic pathways (Zhang et al., 2009). AMPK generally functions as an indispensable heterotrimeric complex that is composed of a catalytic subunit (α1 and α2) and two regulatory subunits (β and γ; Carling, 2004). The direct metabolic substrates of AMPK are related to almost all branches of cellular metabolism (Hoffman et al., 2015; Schaffer et al., 2015). AMPK is widely implicated in diverse biological processes including cell growth, cell polarity and migration, autophagy and energy metabolism (Mihaylova and Shaw, 2011). Importantly, AMPK plays a critical role in regulating fatty acid metabolism, thermogenesis and the development of adipose tissue (O'Neill et al., 2013; Day et al., 2017). AMPK activation is known to inhibit fatty acid synthesis and to promote fatty acid oxidation by phosphorylating acetyl-CoA carboxylase (ACC) and by decreasing malonyl-CoA level, thereby reinstating the activity of carnitine palmitoyltransferase 1 (CPT1) (Carlson and Kim, 1973; Carling et al., 1987; Fullerton et al., 2013). Acute treatment with A-769662, an allosteric AMPK activator that depends on the existence of AMPK β1, lowers the liver malonyl-CoA level and enhances fatty acid oxidation in Sprague Dawley rats, and chronic A-769662 treatment decreases plasma and liver triglyceride levels in *ob/ob* mice (Cool et al., 2006). AMPK also regulates mitochondria biogenesis by phosphorylating and activating PGC-1α (Jager et al., 2007). The natural compound berberine has been shown to promote thermogenesis in brown and WAT via the AMPK-PGC-1α pathway (Zhang et al., 2014). Regarding the development of adipose tissue, several studies have suggested that AMPK plays an inhibitory role in white adipocyte differentiation (Habinowski and Witters, 2001; Dagon et al., 2006; Zhou et al., 2009). Some other studies have shown that AMPK activation promotes brown adipocyte differentiation *in vitro* and that AMPK positively regulates brown adipogenesis and BAT development via epigenetically decreasing the DNA methylation of the PRDM16 promotor (Yang et al., 2016). Most studies on adipocyte AMPK are mainly based on the use of indirect pharmacological AMPK activators, which may cause off-target effects (Cool et al., 2006). For example, pharmacological, chronic activation of AMPK by 5-aminoimidazole-4-carboxamide-1-β-D-ribofuranoside (AICAR) has been reported to enhance energy dissipation in white adipocytes (Gaidhu et al., 2009). When AICAR is converted to 5-aminoimidazole-4-carboxamide ribonucleoside monophosphate (ZMP), it mimics the effects of AMP and activates AMPK (Corton et al., 1995; Carling et al., 2012). However, this mechanism causes other effects, such as the stimulation of glycogen phosphorylase and inhibition of fructose-1,6-bisphosphatase (Cool et al., 2006), and it is unclear whether the metabolic effects seen with AICAR administration are mediated entirely through AMPK stimulation. Therefore, alternative methods for directly and more specifically activating AMPK are needed to study the role of AMPK in adipose metabolism.

Recently, a study reported that adipose tissue-specific deletion of both AMPK β1 and β2 subunits exacerbated high-fat diet (HFD)-induced insulin resistance and hepatic steatosis due to compromised BAT and WAT function (Mottillo et al., 2016). Meanwhile, another study showed that genetic deletion of both AMPK α1 and α2 subunits in adipose reduced adiposity due to an increase in lipolysis and fatty acid oxidation in adipose tissue (Kim et al., 2016). Despite the different genetic knockout strategies used in these two studies, the phenotypes in genotypes with adipocyte AMPK abrogation are quite controversial. In this study, we focus on elucidating the role of AMPK in adipose tissue metabolism by generating a mouse model with the adipocyte AMPK catalytic subunits (α1 and α2) ablated (AKO) and by investigating the metabolic effects of the chronic, direct activation of AMPK by A-769662 on a HFD-fed obese mouse model. Similar to adipose tissue-specific double AMPK β1/β1 KO mice (Mottillo et al., 2016), the AKO mice were prone to HFD-induced obesity and hepatic steatosis and fibrosis, and displayed impaired glucose and lipid metabolism. Consistent with this result, cold-induced adaptive thermogenesis and both basal and β3-adrenergic-activated energy expenditure were significantly blunted in AKO mice. Furthermore, we demonstrated that chronic AMPK activation by A-769662 reduced body weight gain and WAT expansion in HFD-fed mice. Notably, A-769662 enhanced cold-induced thermogenesis and induced browning in the inguinal fat depot. Collectively, our findings indicate that AMPK plays a critical role in the regulation of energy homeostasis and chronic AMPK activation may provide promising therapeutics for treating of obesity and related metabolic diseases through promoting energy expenditure.

## Materials and methods

### Materials

Antibody sources are as follows: UCP-1 (alpha diagnostic, UCP11-A, 1:1,000, 32 kDa), PGC-1a (Calbiochem, ST1202, 1:1,000, 113 kDa); IR (Santa Cruz, sc-711, 1:1,000, 95 kDa), AMPKα1 (#ab3759, 1:1,000, 63 kDa), AMPKα2 (#ab3760, 1:1,000, 63 kDa) (abcam); AMPKγ2 (#AP51709, 1:1000, 38 kDa), β-actin (AM1021B, 1:10,000, 42 kDa) (Abgent); AMPKα (#2532, 1:1,000, 62 kDa), AMPKβ1 (#12063, 1:1,000, 38 kDa), AMPKβ2 (#4148, 1:1,000, 30 kDa), AMPKγ2 (#2536, 1:1,000, 75 kDa), phospho-AMPKα (Thr172) (#2535, 1:1,000, 62 kDa), ACC (#3662, 1:1,000, 280 kDa), phospho-ACC (Ser79) (#3661, 1:1,000, 280 kDa), AKT (#4691, 1:1,000, 60 kDa), phospho-AKT (Ser473) (#4060, 1:1,000, 60 kDa), phospho-IR (Tyr1162) (#3918, 1:1,000, 95 kDa) (Cell Signaling Technology). A-769662, CL 316,243, norepinephrine, rosiglitazone, dexamethasone, 3-Isobutyl-1-methylxanthine (IBMX), 3,3′,5-Triiodo-L-thyronine (T3), indomycine, oligomycin, carbonyl cyanide 4-(trifluoromethoxy) phenylhydrazone (FCCP), rotenone and antimycin A were purchased from Sigma-Aldrich. Recombinant human Insulin (Eli Lily) was purchased from Changzheng Hospital (Shanghai, China). ELISA kits used in measurement of plasma parameters are as follows: TG (Shanghai Fosun Long March, 1.02.1801), TC (Shanghai Fosun Long March, 1.02.0401), HDL-C (XinJianKangCheng Bio, E0303), LDL-C (XinJianKangCheng Bio, E0403), NFEA (WAKO, 294-63601), Insulin (Millipore, EZRMI-13K), Irisin (Phoenix, EK-067-29), Leptin (Millipore, EZML-82K), Adiponectin (abcam, ab108785), Glucagon (BIOSWAMP, MU30638), Epinephrine (CUSABIO, CSB-E08679m), Norepinephrine (CUSABIO, CSB-E07870m), ALT (Sysmex, 290703, 290704), AST (Sysmex, 290705, 290706).

### Animal model

All animal experiments were approved by the Animal Care and Use Committee of the Shanghai Institute of Materia Medica, where the experiments were conducted. All animals were housed in a temperature-controlled room (22 ± 2°C) with a light/dark cycle of 12 h. To obtain adipose tissue-specific AMPKα1/α2 double-KO mice (referred to as AKO mice), AMPKα1/α2-floxed mice were first generated by mating homozygous AMPKα1-floxed mice (stock No: 014141, Prkaa1^fl^, Jackson Laboratory, Bar Harbor, Maine, USA) with AMPKα2-floxed (stock No: 014142, Prkaa2^fl^, Jackson Laboratory, Bar Harbor, Maine, USA). Next, AMPK α1/α2-floxed mice were crossed with Adiponectin-Cre mice (stock No: 010803, Jackson Laboratory, Bar Harbor, Maine, USA) to generate adipose tissue-specific AKO mice. Male AKO mice and age-matched AMPKα1/α2-floxed littermates were randomly divided into two groups and starting from 8 weeks of age for 34 weeks fed either a normal chow diet or a HFD (60% calories from fat, 20% calories from protein, 20% calories from carbohydrate; Research Diets). Body weight and food intake were recorded weekly. Cold exposure experiments were performed at 8 weeks of age. Glucose tolerance tests and insulin tolerance tests were conducted at 20 and 30 weeks of age, respectively. Metabolic analysis and body composition analysis were performed at 40 weeks of age. For chronic anti-obesity studies, beginning at 6 weeks of age, male C57BL/6J mice (Shanghai SLAC Laboratory Animal Co., Shanghai, China) were fed a HFD. At 14 weeks of age, HFD-fed mice and chow-fed mice were randomly assigned to treatment groups. Mice received either vehicle [1% DMSO, 2% castor oil and 0.9% NaCl, q.d., intraperitoneally (i.p.)] or A-769662 (30 mg/kg/day q.d. i.p.) for 6 weeks. Body weight and food intake were recorded daily. Glucose tolerance tests and calorimetry metabolic analysis were conducted during the 4th week of treatment. The blood samples were collected during the 5th week of treatment and the plasma parameters were determined using the indicated kits according to the manufacturers' instructions. Cold exposure experiments were performed at 4°C during the 6th week of treatment. At the end of the study, the tissues were dissected, weighed, immediately frozen in liquid nitrogen and stored at −80°C.

### Cold exposure

For chronic cold exposure, mice were singly housed at 4°C for 24 h. Food and water were available *ad libitum*. For acute cold exposure, mice were individually housed at 4°C for 8 h without food but with free access to water. Body temperature was measured every hour with a BAT-12 microprobe digital thermometer and RET-3 mouse rectal probe (Physitemp Instruments, Clifton, USA).

### Metabolic analysis

Mouse O_2_ consumption, CO_2_ production, heat production and locomotor activity were measured using a sixteen-chamber indirect calorimeter (TSE PhenoMaster, TSE system) according to manufacturer's instructions. Mice were acclimated to the chambers for 24 h before the measurements began. Food and water were fed *ad libitum* throughout the experiment. Basal metabolic parameters were measured during the following 12-h light/dark cycle and the CL 312,643-stimulated metabolic parameters were measured for 10 h after the i.p. injection of CL 312,643 (1 mg/kg). Whole-body fat mass, lean mass and fluid mass were determined by ^1^H-nuclear magnetic resonance (NMR) spectroscopy (Minispec LF90 II, Bruker).

### Glucose tolerance test (GTT) and insulin tolerance test (ITT)

After fasting for 6 h, mice were i.p. injected with either glucose (2 g/kg) or insulin (0.75 U/kg). Glucose concentrations were measured before and 15, 30, 60, 90, and 120 min after the injection of glucose or insulin.

### Quantitative RT-PCR

Total RNA was isolated from cells or homogenized tissues using TRIzol reagent (Invitrogen). One microgram of total RNA was reverse transcribed using PrimeScript Reverse Transcriptase (Takara). The resulting cDNAs were amplified using 2 × SYBR Green qPCR Master Mix (Vazyme) and a Stratagene Mx3005P instrument (Agilent Technologies). Expression was normalized to that of indicated control gene. Primer sequence details are shown in Supplementary Table 1.

### Mitochondrial DNA (mtDNA) quantification

Adipose tissues were cut into small pieces, and total DNA was extracted using a DNeasy Blood & Tissue Kit (Qiagen, 69506) according to the manufacturer's instructions. Quantitative (Q) PCR was performed using mitochondrial DNA-specific primer (16S rRNA)and genomic DNA-specific primer (hexokinase 2 gene, intron 9). Primer sequence details are shown in Supplementary Table 1.

### Immunoblotting

Total protein from the tissues or cells was prepared in RIPA buffer (50 mM Tris-HCl (pH 8.0), 150 mM NaCl, 1% NP-40, 1 mM Na_3_VO_4_, 1 mM DTT, 1 mM EDTA, and 1 mM EGTA) containing complete protease inhibitors (Roche). After boiling for 10 min in SDS loading buffer, equal amounts of protein for each sample were electrophoresed through SDS-PAGE gels.

### Histology

Mouse tissues were fixed in 4% neutral-buffered formalin and embedded in paraffin. Sections (5-μm thick) were stained with hematoxylin and eosin (H&E) and Sirius red according to standard protocols. Microscopy analysis was performed by using a Leica DM6 B microscope at the indicated magnification, and images were captured by a sCMOS camera under the same parameter setting. The average adipocyte size and lipid droplet area in adipose tissue sections [expressed as the average cross-sectional area per cell (μm^2^)] was determined by using ImageJ software (National Institutes of Health) according to the method described in Parlee et al. (2014). Fibrosis was evaluated by calculating the proportional area of picrosirius red-stained matrix using image analysis (Quant center, 3D HISTECH, Hungary).

### Tissue hydroxyproline measurement

Frozen liver samples (100–105 mg) were weighed and acid-hydrolyzed with 5M HCl at 110°C for 18–22 h. Hydroxyproline contents were measured using a hydroxyproline colorimetric assay kit (BioVison, K555-100) according to the manufacturer's instructions.

### Liver triglyceride and cholesterol measurement

The liver triacylglycerol and cholesterol content were measured following a Folch extraction (Folch et al., 1957). The dried lipid residues were then resuspended in 800 μl ethanol with 1% triton for follow-up TG and TC assays. The Liver TG and TC levels were determined with the same kit as used in the plasma analysis.

### Transmission electron microscopy

The adipose tissues were cut into 1 mm^3^ and fixed in 2.5% glutaraldehyde (pH 7.4) for 24 h. Then the sample was washed with 0.1 M phosphate buffer for three times and fixed in osmic acid for 3 h in 4°C. The sample was flushed again, dehydrated with ethanol step by step, and displaced with epoxy propane. Finally, the block was embedded in Spurr resin (Spi-Chem, USA) and polymerization at 70°C. Thin sections were cut on a Leica EM UC6 ultramicrotome and counter stained with uranyl acetate and lead citrate. Then samples were observed with a JEM1230 transmission electron microscope (JEOL, Japan). For each sample, total mitochondria and mitochondria bearing cristae disruption were quantified, and percentage of mitochondria with disrupted cristae was calculated. Criteria for disrupted cristae included any observable disorganization, vacuolization, or dissolution of cristae within mitochondria (Mottillo et al., 2016; Bal et al., 2017a).

### Stromal vascular fraction (SVF) cells isolation and differentiation

SVF cells were isolated as described previously (Wang et al., 2013). In brief, adipose tissue was minced and digested with 10 mg/ml collagenase D (Roche) and 2.4 mg/ml Dispase II (Roche) in PBS supplemented with 1% bovine serum albumin for 45 min at 37°C, followed by quenching with complete medium. The digested tissue suspensions were centrifuged, washed and successively filtered through 100 and 40 μm strainer (BD Biosciences), and then, the cells were plated onto 10 cm dishes. SVFs were cultured in DMEM/F12 supplemented with 10% FBS (Gibco), 1% penicillin/streptomycin (Invitrogen). SVF cells were plated onto 24-well plates to reach confluence. Once cells reached confluency, adipocyte differentiation was carried out in growth medium supplemented with 850 nM insulin, 0.5 mM IBMX, 1 μM dexamethasone, 125 nM indomethacin and 1 nM T3 for 48 h and then in growth medium supplemented with 850 nM insulin and 1 nM T3 for an additional 6 days. To investigate the effect of AMPKα knockdown on NE-induced thermogenic, iWAT-SVF cells were isolated from 5 week-old AMPK α1/α2-floxed mice and induced to differentiate toward beige adipocytes. Cells were infected with NC and Cre adenovirus on day 6 and were treated with NE (10 μM) on day 8 for 6 h. To investigate the effect of A-769662 on thermogenesis, differentiated SVF cells (day 7) were cultured with serum-free DMEM/F12 medium for 2 h and treated with DMSO or A-769662 at the indicated concentration for 6 h in quantitatvie RT-PCR, for 12 h in the measurement of oxygen consumption rates (OCRs) and for 24 h in western blot analysis. To confirm whether A-769662-induced thermogenesis *in vitro* via AMPK signaling pathway, iWAT-SVF cells isolated from the floxed mice were differentiated into beige adipocytes followed by infected with NC and Cre lentivirus on day 6, and were treated with A-769662 at the indicated concentration on day 8.

### Measurement of OCRs

SVF cells were plated in a 96-well XF microplate (Seahorse Bioscience) and differentiated for 7 days, OCR was measured at 37°C using a 96 Extracellular Flux Analyzer (Seahorse Bioscience) in accordance with the manufacturer's instructions. Uncoupled respiration was detected by treating cells with oligomycin (2 μM).

### Tissue distribution assay

HFD-fed mice were i.p. injected with A-769662 (30 mg/kg). After 1 h, the animals were sacrificed, and the plasma, liver, and various adipose depots and muscles were collected and preserved at −80°C. Tissue samples were analyzed with a liquid chromatography-mass spectrometry/mass spectrometry (LC-MS/MS) system (an Agilent 1200 HPLC coupled to an Agilent 6460 Triple Quad instrument, Agilent Technologies, USA) to detect the concentration of A-769662. Data were analyzed by MassHunter Quantitative Analysis (version B.02.01, Agilent Technologies, USA).

### Statistical analysis

The results are presented as the means ± SEM. Differences between the groups were analyzed using Student's *t*-test or one-way ANOVA followed by Dunnett's multiple comparisons test by GraphPad Prism version 7.00 for Windows (GraphPad Software, La Jolla California USA). *P* < 0.05 was regarded as statistically significant.

## Results

### Adipocyte AMPK regulating mitochondrial biogenesis and structural integrity was required for cold-induced adaptive thermogenesis in inguinal white adipose tissue

**Figure 1:**
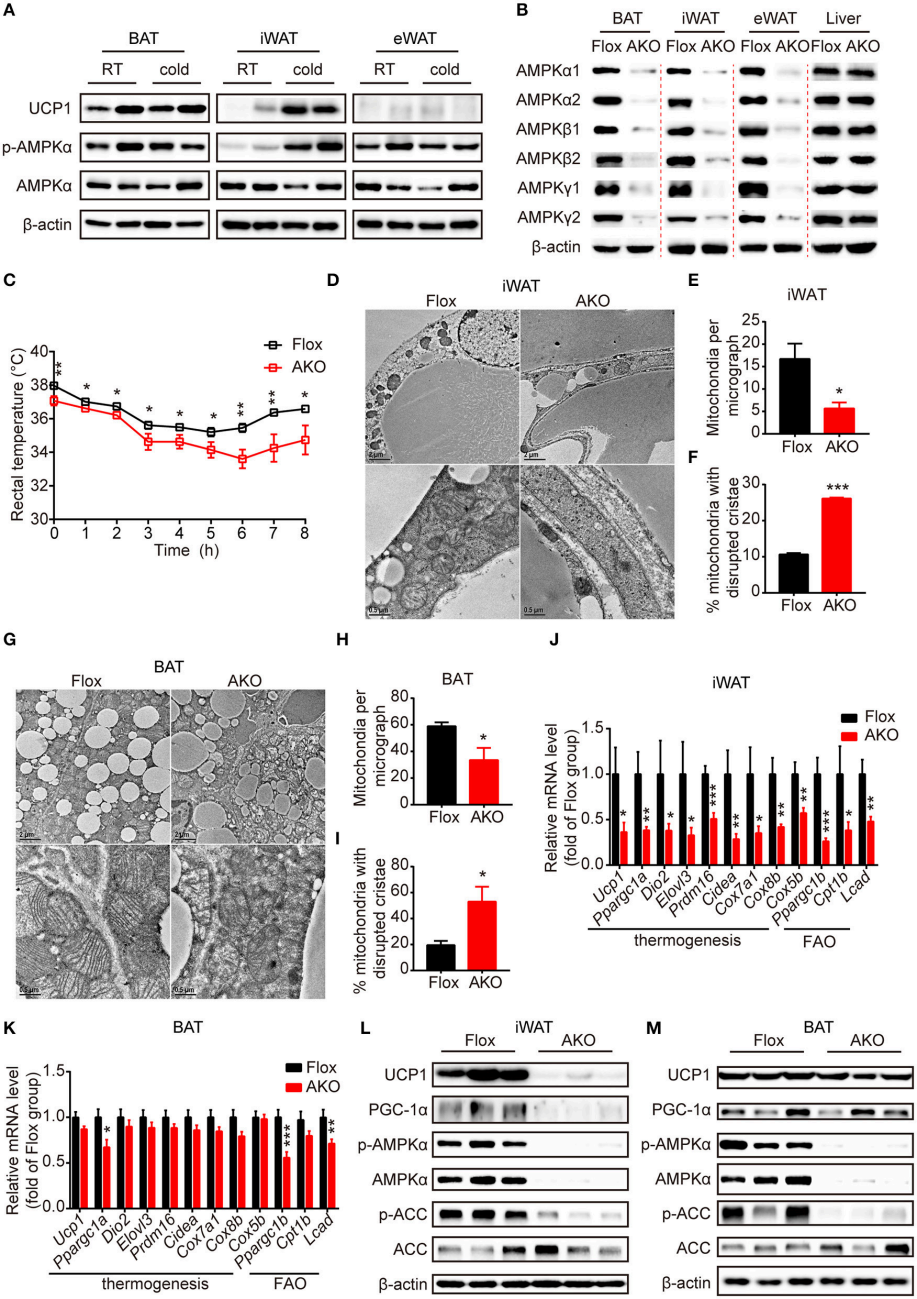
Adipose AMPK deficiency impaired cold tolerance and suppressed cold-induced thermogenesis specifically in inguinal white adipose tissue. **(A)** Western blot analysis of UCP1, p-AMPKα (T172) and AMPKα protein levels in the interscapular brown adipose tissue (BAT), inguinal white adipose tissue (iWAT) and epididymal white adipose tissue (eWAT) of 9-week-old male wild-type (WT) mice housed at RT or 4°C for 24 h. β-actin was used as a loading control. *n* = 4. **(B)** Western blot analysis of AMPKα1, α2, β1, β2, γ1, γ2 protein expression levels in the BAT, iWAT, eWAT and liver of 10-week-old male AKO mice and age-matched floxed littermates. β-actin was used as a loading control. *n* = 3. **(C)** Rectal temperature of 8-week-old chow-fed AKO and floxed mice at 4°C for 8 h. *n* = 9. **(D–M)** 8-week-old male chow-fed AKO mice and floxed mice were housed at 4°C for 48 h. Representative transmission electronic microscopy images of iWAT **(D)** and BAT **(G)**, total number of mitochondria per micrograph in iWAT **(E)** and BAT **(H)** and the percentage of mitochondria with disrupted cristae over total mitochondria in iWAT **(F)** and BAT **(I)** from AKO mice and floxed mice were shown. *n* = 3. Scale bar: 2 μm in low magnification (×5,000, upper) and 0.5 μm in high magnification (×20,000, bottom). The relative mRNA levels of thermogenic genes and fatty acid oxidation (FAO)-related genes in the iWAT **(J)** and BAT **(K)** of AKO mice and floxed mice were analyzed by quantitative RT-PCR (normalized to *36b4*). *n* = 8–9. The expression levels of AMPKα, p-AMPKα (T172), ACC, p-ACC (S79), UCP1 and PGC-1α in the iWAT **(L)** and BAT **(M)** of AKO mice and floxed mice were determined by western blot analysis. Data are presented as the means ± SEM. Student's *t*-test. ^*^P < 0.05, ^**^P < 0.01, ^***^P < 0.001 compared with the indicated control group.

One of the important functions of brown and beige fat is defending against hypothermia in cold environments through adaptive thermogenesis, which is crucial for maintaining whole-body energy homeostasis (Harms and Seale, 2013). Cold exposure stimulates β-adrenergic signaling via regulating norepinephrine (NE) secretion from sympathetic nerves, which indirectly activates AMPK signaling by inducing lipolysis and mitochondrial uncoupling in the adipose tissue of rodents (Gauthier et al., 2008). It has been reported that in response to cold acclimation, different adipose tissue depots play diverse roles in metabolic remodeling, displaying enhanced thermogenic activity in BAT and contributing to browning in iWAT and in eWAT to a lesser extent (Jia et al., 2016). We wondered whether the sensitivity of AMPK activation in response to short-term cold exposure varied among different fat pads. To probe the role of AMPK signaling in cold-induced adaptive thermogenesis in different adipose depots, 9-week-old male C57BL/6J mice were housed at room temperature (RT) or challenged with cold temperature (4°C) for 24 h, and the expression levels of proteins reflecting AMPK activation and thermogenic capacity in various adipose depots, including interscapular BAT, inguinal white adipose tissue (iWAT), and epididymal white adipose tissue (eWAT), were examined by western blot analysis. AMPK was significantly activated by cold exposure in iWAT, as evidenced by increased AMPKα phosphorylation (Thr172), but was unaffected in BAT and eWAT (Figure 1A and Supplementary Figure 1A). Similarly, the expression of the thermogenic protein UCP1 was up-regulated by cold in iWAT, but not in BAT and eWAT (Figure 1A and Supplementary Figures 1A,B), suggesting a depot-specific positive correlation between AMPK activation and adaptive thermogenesis in iWAT.

To investigate the physiological effects of adipocyte AMPKα on whole-body energy metabolism, we generated adipose tissue-specific AMPKα knockout (referred to as AKO) mice by crossing Adiponectin-Cre mice with double AMPK α1/α2-floxed mice (referred to as floxed mice). Western blot analyses of AMPKα1 and AMPKα2 expression in different fat pads and liver were performed to examine the knockout efficiency of AKO mice. The expression of both AMPKα1 and AMPKα2 and the mRNA levels of their encoding genes (*Prkaa1* and *Prkaa2*) in iWAT, eWAT, and BAT were substantially reduced in AKO mice; in contrast, that in the liver was not different between the genotypes (Figure 1B and Supplementary Figures 1C,D,I–L), indicating that the deletion of AMPKα was specific to adipose tissue. Because AMPKα function as an indispensable subunit of the heterotrimeric complex (Fentz et al., 2015), we examined the expression levels of its regulatory subunits β and γ. Surprisingly, expression of subunit AMPKβ1, AMPKβ2, AMPKγ1, and AMPKγ2 were significantly decreased in adipose tissue of AKO mice, but not in liver (Figure 1B and Supplementary Figures 1E–H). However, the mRNA levels of genes encoding subunit β (*Prkab1* and *Prkab2*) and subunit γ (*Prkag1* and *Prkag2*) were unaltered between genotypes (Supplementary Figures 1I–L), which suggests that the presence of AMPKα might be critical for protein stability of β subunits and γ subunits. These data indicate that efficient adipocyte-specific deletion of AMPK was achieved.

First, chow-fed male AKO mice and age-matched floxed littermates were challenged with cold exposure at 4°C for 8 h to assess the cold tolerance of both genotypes. During the cold challenge, the rectal temperature of AKO mice decreased more rapidly than that of floxed mice (Figure 1C), indicating that the capacity of adaptive thermogenesis in AKO mice was impaired. To determine the adaptive thermogenic activity of different adipose pads, cold exposure was extended to 48 h. The content and integrity of mitochondria affect its function of oxidation metabolism and cellular energy status during thermogenesis process (Bal et al., 2017a). The total number of mitochondria in different adipose pads was determined by assessing mitochondrial DNA copy number. The mitochondria number was reduced in iWAT and BAT of AKO mice compared to that of floxed mice, whereas that in eWAT did not differ in these two genotypes (Supplementary Figure 1M), suggesting that AMPK deletion likely inhibited mitochondrial biogenesis in iWAT and BAT, both of which contribute to adaptive thermogenesis during prolonged cold exposure. Next, the mitochondrial morphology in iWAT and BAT were assessed by using transmission electron microscopy. Accordingly, the mitochondria number per micrograph was decreased in iWAT and BAT of AKO mice by approximately 66 and 43%, respectively (Figures 1D,E,G,H). The mitochondrial structure was altered in iWAT and BAT of AKO mice after cold adaption and the disrupted cristae were increased by approximately 146 and 170%, respectively (Figures 1D,F,G,I). These observations indicate that adipocyte AMPK deficiency impaired both the structural integrity of mitochondria and mitochondrial biogenesis.

In addition, the expression of thermogenesis-related genes and proteins responding to cold acclimation was detected by Quantitative RT-PCR. The mRNA levels of thermogenesis-related genes, including *Ucp1, Ppargc1a, Dio2, Elovl3, Prdm16, Cidea, Cox7a1, Cox8b*, and *Cox5b*, and fatty acid oxidation (FAO)-related genes, such as *Ppargc1b, Cpt1b*, and *Lcad*, were dramatically reduced in the iWAT of the AKO mice after cold exposure (Figure 1J), but were unchanged in eWAT (Supplementary Figure 1P). In agreement of this result, the expression levels of the thermogenic protein UCP1 and of PGC-1α, a master coregulatory factor for mitochondrial biogenesis (Wu et al., 1999), were significantly reduced by genetic deletion of adipocyte AMPKα (Figure 1L and Supplementary Figure 1N), indicating that cold-induced browning was severely impaired in the iWAT of AKO mice. In addition to the thermogenic remodeling of iWAT, BAT-mediated adaptive thermogenesis is also highly responsive to cold stimulation (Rosen and Spiegelman, 2014). We then examined the expression pattern of thermogenic genes and proteins in the BAT of cold-acclimated AKO mice and floxed mice. In line with the reduced content and disrupted cristae of mitochondria, the expression of *Ppargc1a* and FAO-related genes, including *Ppargc1b* and *Lcad*, were decreased in BAT of AKO mice. However, the expression of thermogenic genes, such as *Ucp1, Ppargc1a, Dio2, Elovl3, Prdm16, Cidea, Cox7a1, Cox8b*, and *Cox5b*, were not significantly altered in the BAT of AKO mice (Figure 1K). Western blot analysis showed that the expression of UCP1 and PGC-1α in the BAT of the AKO mice was comparable with that of the floxed mice (Figure 1M and Supplementary Figure 1O), denoting that BAT-mediated adaptive thermogenesis was not readily perturbed by adipocyte AMPKα deletion *in vivo*. Moreover, the knockdown of AMPKα by using Cre-expressing adenovirus in differentiated stromal vascular fraction (SVF) cells isolated from the iWAT of AMPK α1/α2-floxed mice inhibited NE-induced thermogenic gene induction (Supplementary Figure 2), indicating that AMPK may serve as an important modulator of thermogenesis in a cell-autonomous manner. Together, these data demonstrate that the abrogation of AMPKα in adipose tissue impairs mitochondrial integrity and function and suppresses cold-induced adaptive thermogenesis and the browning of white adipocytes in iWAT, which may contribute to cold intolerance of AKO mice.

### Adipocyte AMPK protected against diet-induced obesity and related metabolic dysfunction

**Figure 2:**
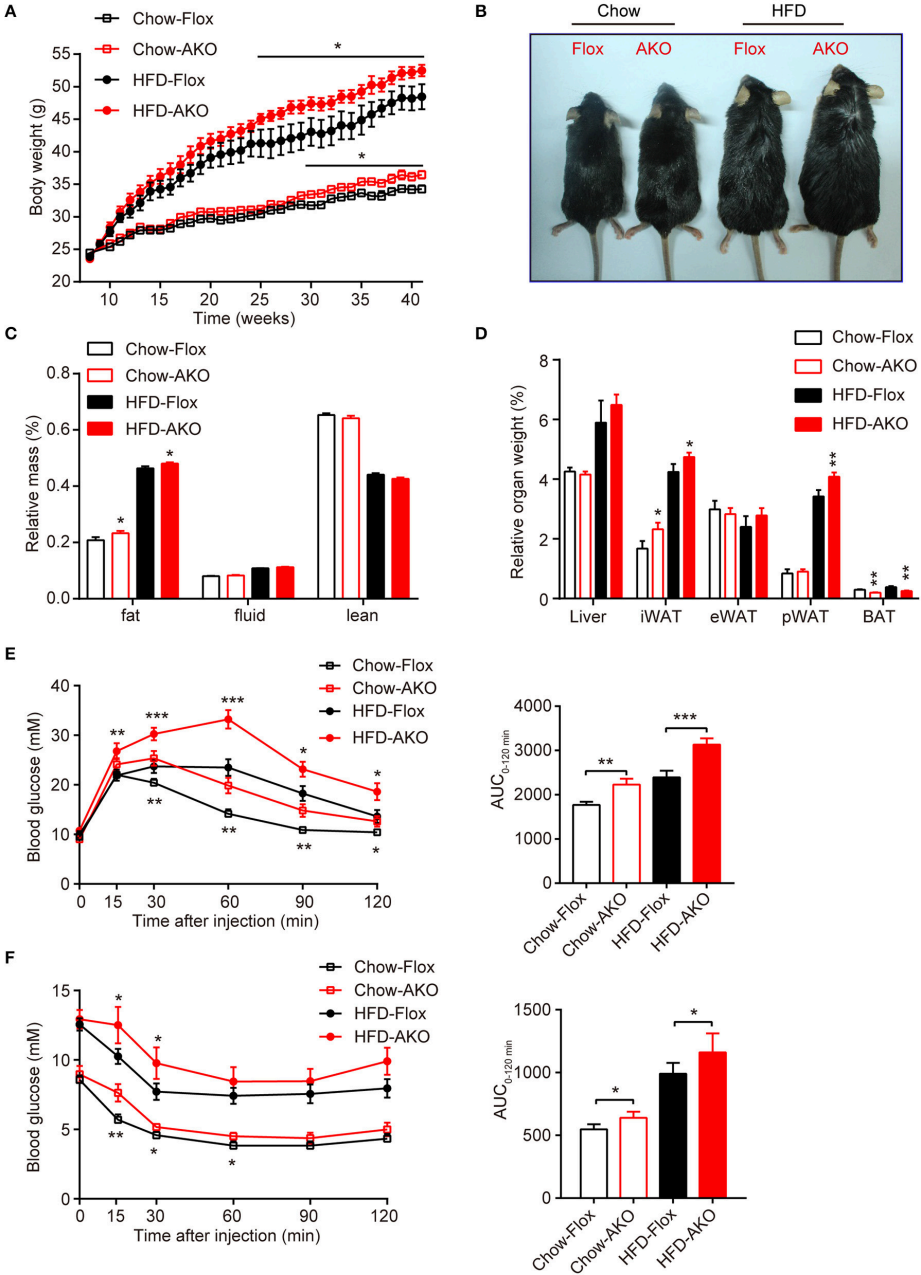
Adipose-specific AMPKα deletion exacerbated high-fat diet (HFD)-induced adiposity and glucose intolerance. **(A)** Change in body weight of AKO mice and age-matched floxed littermates during the indicated period. **(B)** Representative images of 41-week-old male AKO mice and age-matched flox/flox mice. **(C)** Fat mass, fluid mass and lean mass relative to the total body weight of 40-week-old mice. **(D)** Relative weight of the liver, iWAT, eWAT, pWAT, and BAT to the total body weight of mice. **(E)** Blood glucose levels from the intraperitoneal glucose tolerance test (ipGTT) of 20-week-old mice following a single injection of glucose (2 g/kg) (left). The area under the curve (AUC) of the ipGTT during the indicated times was calculated as the blood glucose multiplied by the time (mM^*^min) (right). **(F)** Blood glucose levels of 30-week-old mice following a single injection of insulin (0.75 U/kg) in the intraperitoneal insulin tolerance test (ITT) (left). The AUC of the ITT is shown (right). Chow-Flox: AMPK α1/α2-floxed mice fed a chow diet, Chow-AKO: adipose tissue-specific AMPK α1/α2 KO mice fed a chow diet, HFD-Flox: AMPK α1/α2-floxed mice fed a HFD, HFD-AKO: adipose tissue-specific AMPK α1/α2 KO mice fed a HFD. Data are presented as the means ± SEM. *n* = 11–15. Student's *t*-test. ^*^P < 0.05, ^**^P < 0.01, ^***^P < 0.001 compared with the corresponding Flox group.

To investigate the metabolic effects of adipocyte AMPK ablation in response to a HFD, 8-week-old male AKO mice and age-matched floxed littermates were fed either a chow diet or a HFD for 34 weeks. Beginning at 29 weeks of age, the chow-fed AKO mice gained more weight than their counterparts, and the HFD challenge exacerbated the weight gain caused by the deletion of adipocyte AMPK, as evidenced by the earlier appearance (at 25 weeks of age) of more severe obesity in HFD-fed AKO mice (Figure 2A). Accordingly, the AKO mice were visually bigger and more obese than the HFD-fed or chow-fed floxed mice (Figure 2B). Although adipocyte AMPKα deletion did not affect food intake in mice fed a chow diet or a HFD (Supplementary Figure 3), chow- and HFD-fed AKO mice exhibited greater adiposity than the floxed mice, as evidenced by a higher fat mass in the AKO groups (Figure 2C). The relative iWAT weight was increased in AKO mice fed a chow diet or a HFD, but the weight of perirenal WAT (pWAT) was increased only in the HFD-fed AKO mice, while relative weights of eWAT and liver were unchanged (Figure 2D). Interestingly, the relative weight of BAT was decreased in the chow- and HFD-fed AKO mice, probably due to the inhibition of brown adipogenesis during early BAT development by the deletion of AMPKα (Yang et al., 2016). Moreover, glucose tolerance was impaired in the AKO mice fed a chow diet or a HFD, as shown by an increased area under the curve (AUC) in this group (Figure 2E). Defects in insulin sensitivity were also observed in the AKO mice fed a chow diet or a HFD (Figure 2F). Notably, the plasma levels of insulin and leptin, an adipokine that positively correlates to obesity and fat mass (Considine et al., 1996), were markedly elevated in both chow- and HFD-fed mice, while as an important adipose-derived hormone, adiponectin, the level of which is closely associated with insulin resistance and negatively correlated with adiposity (Yamauchi et al., 2001), was decreased by 36 and 32% in chow- and HFD-fed AKO mice, respectively (Table 1). The plasma level of glucagon, acting as a counterregulatory hormone for insulin (Jiang and Zhang, 2003), was reduced in both chow- and HFD-fed mice (Table 1), which may be a consequence of increased insulin level in AKO mice. Furthermore, the plasma levels of triglyceride (TG) and total cholesterol (TC) were significantly increased by 34 and 39% in HFD-fed AKO mice, respectively, but not in chow-fed AKO mice (Table 1), indicating that adipocyte AMPK deletion aggravated the development of hyperlipidemia under HFD stress. However, there were no significant changes in the levels of low-density lipoprotein cholesterol (LDL-C), high-density lipoprotein cholesterol (HDL-C) and nonesterified fatty acid (NEFA) between genotypes fed with a chow-diet or a HFD (Table 1). Collectively, these results suggest that adipocyte AMPK play a vital role in combating HFD-induced obesity, dysregulated glucose homeostasis and insulin resistance.

**Table 1:** Plasma metabolic variables in floxed mice and AKO mice fed with a chow diet or a HFD.

**Parameters**	**Chow-Flox**	**Chow-AKO**	**HFD-Flox**	**HFD-AKO**
TC (mM)	1.97 ± 0.13	2.01 ± 0.17	4.2 ± 0.47	5.84 ± 0.27^**^
LDL-C (mM)	1.49 ± 0.04	1.60 ± 0.05	2.67 ± 0.21	3.23 ± 0.16
HDL-C (mM)	0.92 ± 0.04	0.84 ± 0.06	1.56 ± 0.11	1.65 ± 0.04
LDL-C/HDL-C	1.65 ± 0.07	1.77 ± 0.06	1.92 ± 0.07	1.81 ± 0.09
TG (mM)	0.70 ± 0.05	0.69 ± 0.05	0.41 ± 0.04	0.55 ± 0.04^**^
NEFA (mEq/L)	0.83 ± 0.06	0.86 ± 0.06	0.54 ± 0.06	0.53 ± 0.03
Insulin (ng/ml)	3.04 ± 0.39	4.34 ± 0.53^*^	20.08 ± 3.56	29.31 ± 3.59^*^
Leptin (ng/ml)	3.40 ± 0.62	5.74 ± 0.81^*^	45.97 ± 4.53	57.08 ± 2.19^*^
Irisin (ng/ml)	55.05 ± 1.74	47.47 ± 5.49	34.64 ± 1.5	33.21 ± 2.13
Adiponectin (μg/ml)	15.20 ± 2.72	9.78 ± 0.88^*^	14.63 ± 1.52	9.99 ± 0.99^*^
Glucagon (pg/ml)	231.67 ± 20.24	147.10 ± 32.72^*^	119.09 ± 15.19	77.18 ± 16.28^*^
Epinephrine (pg/ml)	234.30 ± 40.59	263.74 ± 35.51	235.32 ± 24.75	208.48 ± 30.08
NE (pg/ml)	541.40 ± 5.97	532.86 ± 13.41	501.83 ± 10.87	502.49 ± 11.95

*Values are expressed as the means ± SEM. n = 7–8. Student's t-test*.

* *P < 0.05*,

** *P < 0.01 compared with the corresponding Flox group. TC, total cholesterol; NEFA, nonesterified fatty acid; HDL-C, high-density lipoprotein cholesterol; LDL-C, low-density lipoprotein cholesterol; TG, triglycerides; NE, norepinephrine*.

### Effects of adipocyte AMPKα deficiency on the morphology of adipocytes in different depots *in vivo*

**Figure 3:**
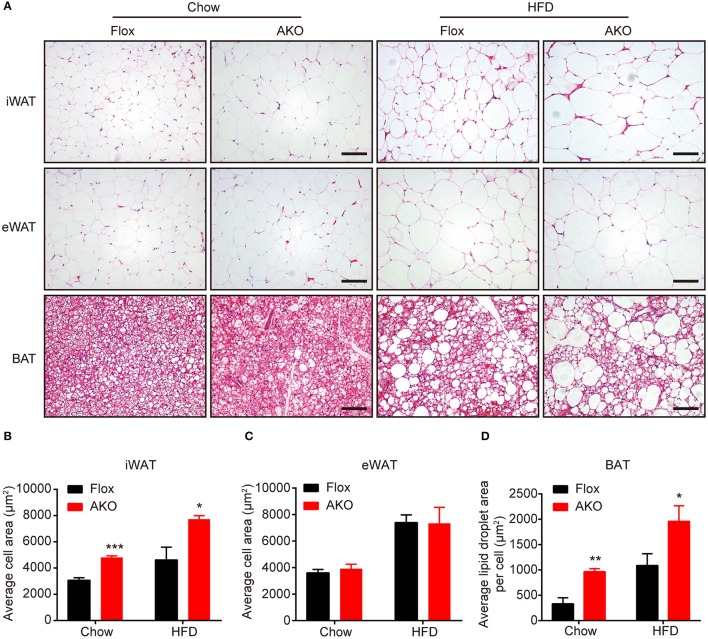
Lack of adipocyte AMPKα increased adipocyte size in inguinal white adipose tissue. **(A)** Representative H&E stained images of iWAT, eWAT, and BAT in AKO mice and age-matched floxed littermates fed a chow diet (left) or a HFD (right). Scale bar = 100 μm. **(B–D)** Average adipocyte area in iWAT **(B)** and eWAT **(C)** and average lipid droplet area in the BAT **(D)** in AKO mice and floxed littermates fed a chow diet or a HFD. Data are presented as the means ± SEM. *n* = 5-7. Student's *t*-test. ^*^P < 0.05, ^**^P < 0.01, ^***^P < 0.001 compared with the corresponding Flox group.

Since the AKO mice were prone to HFD-induced obesity, we further investigate the effect of adipocyte AMPKα deletion on the morphology of different adipose depots. H&E staining showed that adipocyte size in the iWAT of chow- or HFD-fed AKO mice were notably larger than that in the floxed mice, as indicated by increased adipocyte area in the iWAT (Figures 3A,B). However, the adipocyte size in the eWAT of chow- and HFD-fed mice was not influenced by adipocyte AMPKα deletion (Figures 3A,C). The lipid droplets in the BAT of chow- or HFD-fed AKO mice were obviously larger than those of the floxed mice (Figures 3A,D), which may be owing to diminished expression of FAO-related gene in the BAT (Figure 1K). As mentioned above, adipocyte AMPKα deficiency specifically affected the expression of thermogenesis- and FAO-related genes and proteins in iWAT, which may hinder lipid mobilization and contribute to the increased adipocyte size and weight of inguinal adipose tissue.

### Adipocyte AMPKα deficiency reduced basal and β3-adrenergic-activated energy expenditure

**Figure 4:**
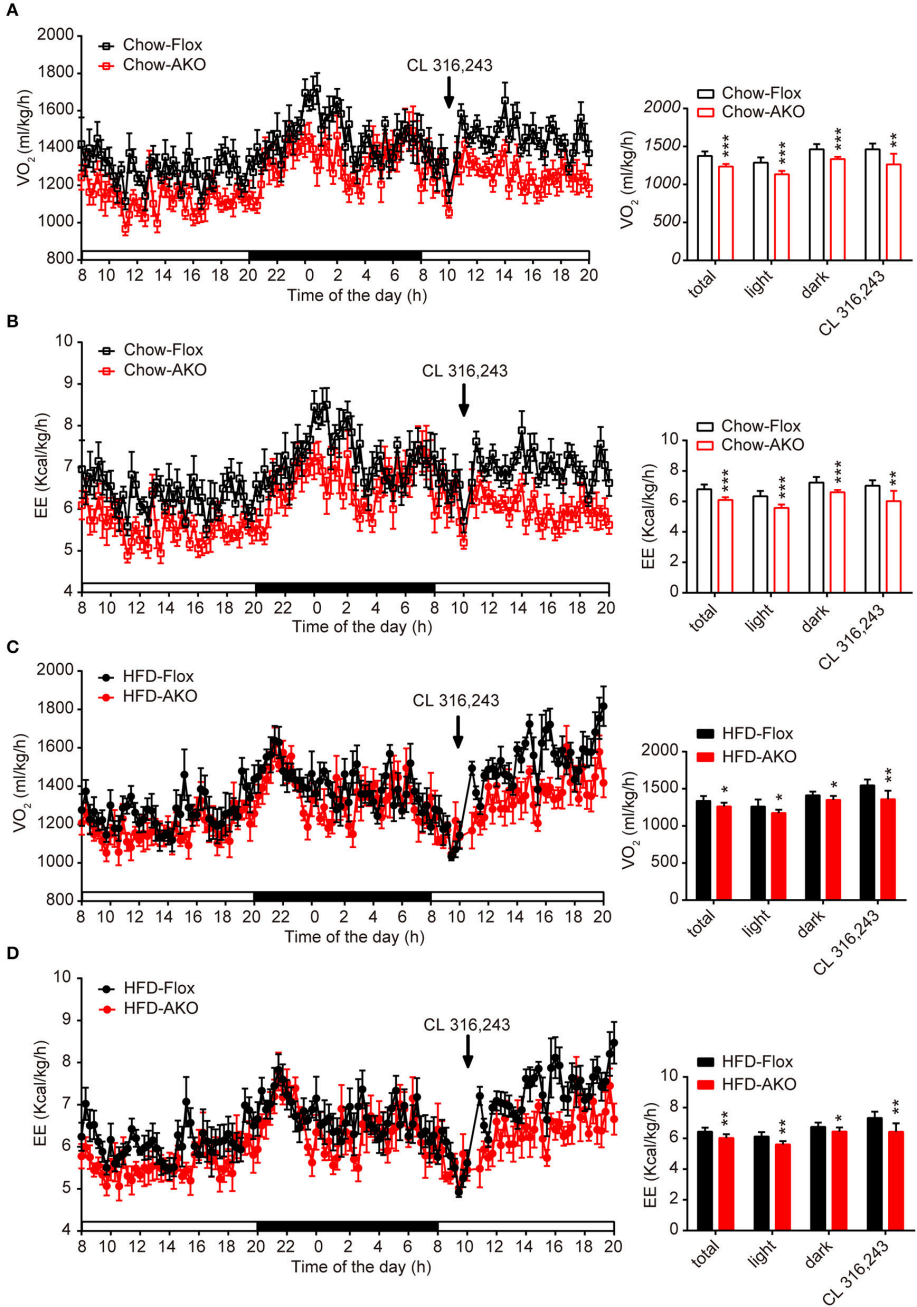
Deletion of adipocyte AMPKα reduced basal and β3-adrenergic-activated energy expenditure in chow- and HFD-fed mice. **(A–D)** Metabolic cage analyses of 40-week-old AKO and floxed mice fed a chow or a HFD were performed. **(A)** The O_2_ consumption of chow-fed mice during a 12-h light-dark cycle and after an injection of CL 312,643 (1 mg/kg) (left). Average basal and CL 312,643-stimulated O_2_ consumption of chow-fed mice (right). **(B)** The energy expenditure (EE) of chow-fed mice during a 12-h light-dark cycle and after an injection of CL 312,643 (1 mg/kg) (left). Average basal and CL 312,643-stimulated EE of chow-fed mice (right). **(C)** The O_2_ consumption of HFD-fed mice during a 12-h light-dark cycle and after an injection of CL 312,643 (1 mg/kg) (left). Average basal and CL 312,643-stimulated VO_2_ consumption of HFD-fed mice (right). **(D)**The EE of HFD-fed mice during a 12-h light-dark cycle and after an injection of CL 312,643 (1 mg/kg) (left). Average basal and CL 312,643-stimulated EE of HFD-fed mice (right). Data are presented as the means ± SEM. *n* = 8. Student's *t*-test. ^*^P < 0.05, ^**^P < 0.01, ^***^P < 0.001 compared with the corresponding Flox group.

Since the development of obesity and obesity-related metabolic dysfunction is generally due to an imbalance between energy intake and energy expenditure, we continued to evaluate the effect of adipocyte AMPKα deletion on energy expenditure under basal conditions and in response to acute β3-adrenergic receptor (AR) activation. Under basal conditions or after i.p. injections with β3-AR activator CL 316,243, the O_2_ consumption and energy expenditure (EE) of chow- and HFD-fed AKO mice were significantly reduced during the 12-h light-dark cycle (Figures 4A–D), but locomotor activity was unchanged (Supplementary Figures 4B,D). Additionally, the basal respiration exchange ratio (RER) was unaltered in the AKO mice fed a chow diet or a HFD (Supplementary Figures 4A,C), but the CL 316,243-stimulated RER was slightly decreased in the AKO mice fed the chow diet (Supplementary Figure 4A). The plasma levels of catecholamines, including epinephrine and norepinephrine, and irisin were unaltered between genotypes (Table 1), which excludes the effects of circulating hormones on energy metabolism of AKO mice. Overall, these data signify that the impaired energy expenditure due to adipocyte AMPKα ablation results in obesity and that adipocyte AMPK is important for the regulation of whole-body energy homeostasis.

### Lack of adipocyte AMPKα promoted the development of liver steatosis and fibrosis

**Figure 5:**
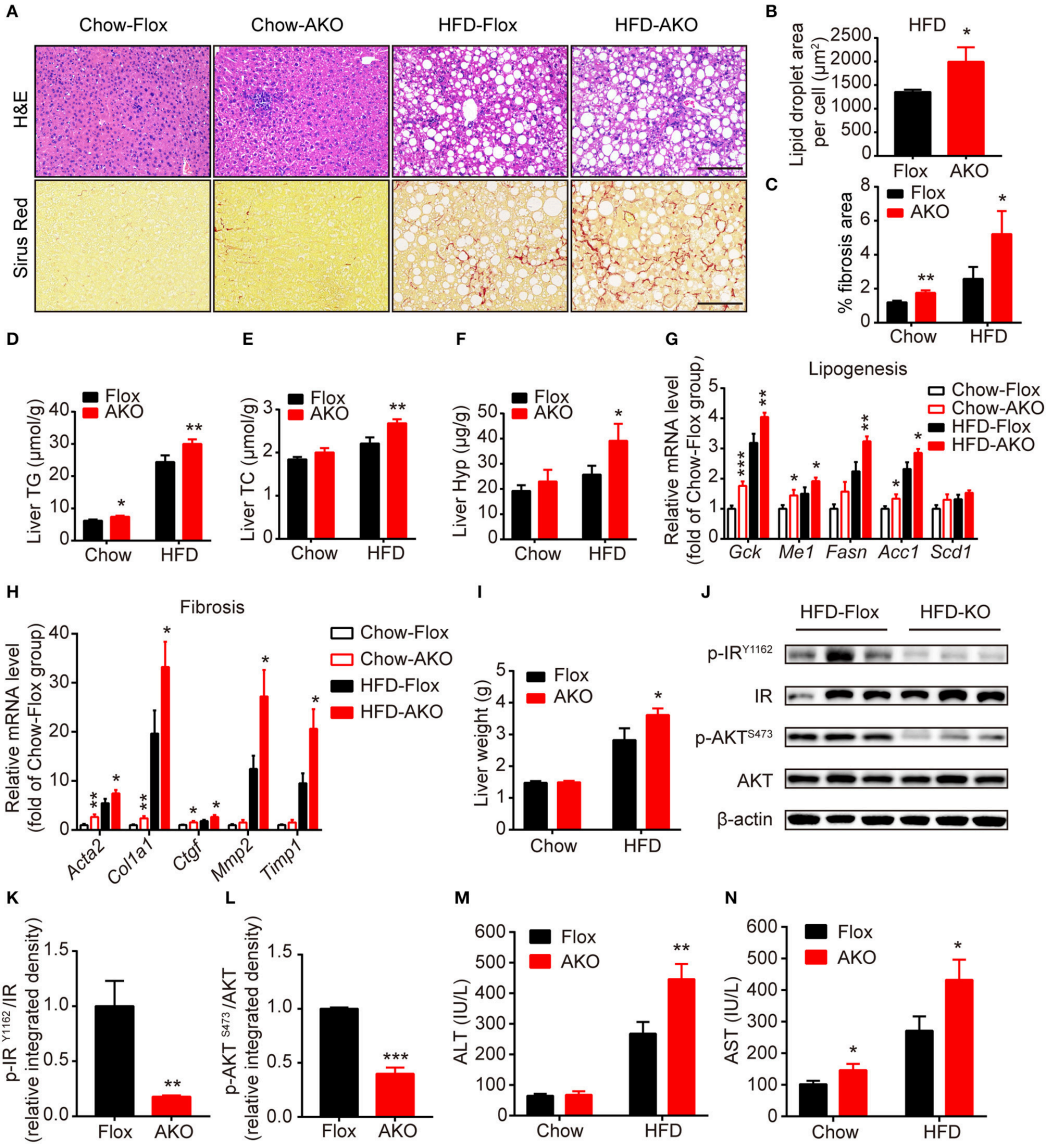
Adipocyte AMPK deficiency promoted the development of liver steatosis and fibrosis. **(A)** Representative H&E stained (top) and Sirius red stained (bottom) images of liver in AKO mice and age-matched floxed littermates fed with a chow diet (left) or a HFD (right). Scale bar = 100 μm. **(B)** Average lipid droplet area per cell in liver in AKO mice and floxed littermates fed with a chow diet or a HFD. **(C)** Fibrosis area in liver were evaluated in chow- or HFD-fed AKO mice and floxed littermates. **(D,E)** Liver TG **(D)** and TC (**E**) levels in chow- or HFD-fed AKO mice and floxed littermates. **(F)** Liver hydroxyproline level in chow- or HFD-fed AKO mice and floxed littermates. **(G,H)** The relative mRNA levels of lipogenesis genes **(G)** and fibrosis-related genes **(H)** in the liver of in chow- or HFD-fed AKO mice and floxed littermates were analyzed by quantitative RT-PCR (normalized to *Actb*). *n* = 8–9. **(I)** Absolute liver weight in chow- or HFD-fed AKO mice and floxed littermates. **(J)** The expression levels of AKT, p-AKT (S473), IR, p-IR (Y1162) in the liver of HFD-fed AKO mice and floxed littermates were determined by western blot analysis. (**K,L**) Relative phosphorylation levels of AKT and IR were determined by densitometric quantification of the immunoblots shown in **(J)**. *n* = 3. **(M,N)** Plasma ALT and AST levels in chow- or HFD-fed AKO mice and floxed littermates. Data are presented as the means ± SEM. Student's *t*-test. ^*^P < 0.05, ^**^P < 0.01, ^***^P < 0.001 compared with the corresponding Flox group.

As it was previously shown that deletion of adipocyte AMPK β1/β2 subunits induces hepatic lipid accumulation and liver insulin resistance (Mottillo et al., 2016), we then examined the effect of loss of adipocyte AMPKα on hepatic metabolism in our genotypes. H&E staining showed that hepatocytes in AKO mice displayed more lipid accumulation with enlarged lipid droplets (Figures 5A,B), additionally evidenced by the increased liver content of TG and TC in HFD-fed AKO mice (Figures 5D,E). Sirus Red staining indicated that liver fibrosis area was augmented in AKO mice fed with a chow diet or HFD (Figures 5A,C) and liver hydroxyproline (main component of collagen) level was significantly increased in HFD-fed AKO mice (Figure 5F), suggesting appearance of severe fibrosis in the liver of HFD-fed AKO mice. In addition, the expression levels of lipogenesis-related genes, such as glucose kinase (*Gck*), malic enzyme (*Me1*), fatty acid synthase (*Fasn*) and acetyl-CoA carboxylase 1 (*Acc1*), and fibrosis-related genes including smooth muscle actin alpha 2 (Acta2, encoding α smooth muscle actin), collagen type I alpha 1 chain (Col1a1), connective tissue growth factor (*Ctgf*), matrix metallopeptidase 2 (*Mmp2*) and tissue inhibitor of metalloproteinases 1 (*Timp1*) were up-regulated in AKO mice especially fed with a HFD (Figures 5G,H). Despite unchanged relative weight of liver between genotypes (Figure 2D), the absolute liver weight was obviously increased in HFD-fed AKO mice (Figure 5I). We further explored the status of insulin resistance in the liver of HFD-fed floxed and AKO mice by detecting the endogenous activity of insulin signaling. The phosphorylation levels of insulin receptor (IR) tyrosine-1162 (IR^Y1162^) and serine/threonine kinase (AKT) serine-473 (AKT^S473^) were dramatically repressed in HFD-fed AKO mice (Figures 5J–L), indicating impairment in insulin sensitivity in the liver after adipocyte AMPKα deletion. Moreover, the plasma levels of alanine aminotransferase (ALT) and aspartate transaminase (AST) were significantly increased in HFD-fed AKO mice (Figures 5M,N), verifying that hepatic lipotoxicity trended to be more severe in AKO mice. Taken together, these results demonstrated that adipocyte AMPKα ablation induced hepatic lipid accumulation and eventually exacerbated the development of liver steatosis and even fibrosis.

### Chronic treatment with the AMPK activator A-769662 alleviated HFD-induced obesity, glucose, and lipid metabolic disorders

**Figure 6:**
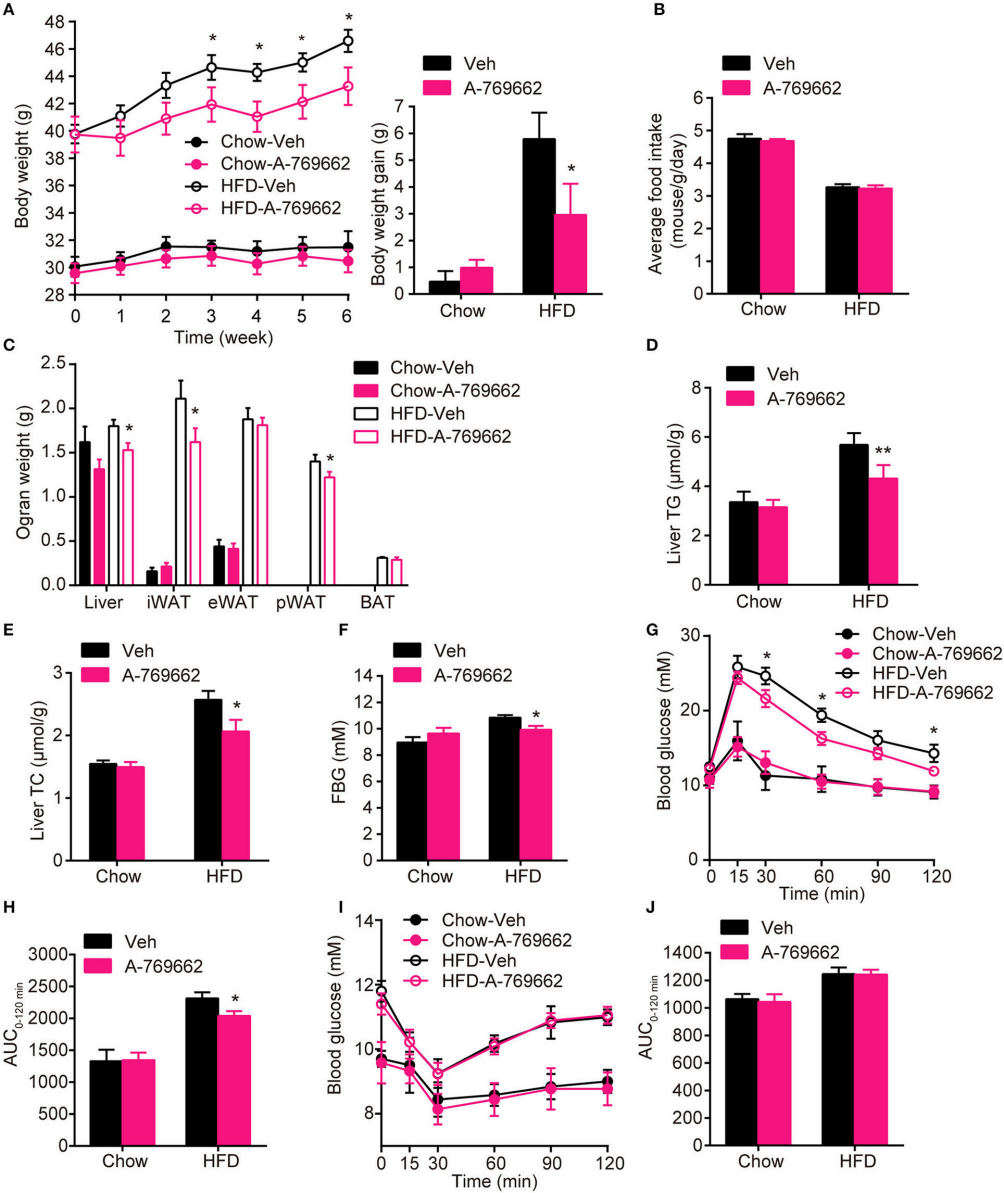
Chronic AMPK activation by A-769662 protected against HFD-induced obesity and dysregulated glucose metabolism **(A)** Body weight (left) and average body weight gain (right) of chow- and HFD-fed mice during the 6-week treatment period. **(B)** Average food intake of chow- and HFD-fed mice during the 6-week treatment period. **(C)** Absolute weights of the liver, iWAT, eWAT, pWAT and BAT of chow- and HFD-fed mice after 6-week treatment. **(D,E)** Liver TG **(D)** and TC (**E**) levels of chow- and HFD-fed mice after 6-week treatment. **(F)** Fasting blood glucose was measured during the 4th week of treatment. **(G,H)** Intraperitoneal glucose tolerance tests of chow- and HFD-fed mice were conducted at week 4 of treatment **(G)**. The AUC from 0 to 120 min was calculated **(H)**. (**I,J**) Insulin tolerance test of chow- and HFD-fed mice were conducted at week 5 of treatment **(I)**. The AUC from 0 to 120 min was calculated **(J)**. Chow-Veh: mice fed a chow diet and treated with vehicle, Chow-A-769662: mice fed a chow diet and treated with A-769662, HFD-Veh: mice fed a HFD and treated with vehicle, HFD-A-769662: mice fed a HFD and treated with A-769662. Data are presented as the means ± SEM. *n* = 5–9. Student's *t*-test. ^*^P < 0.05, ^**^P < 0.01 compared with the indicated control group.

AMPK activity has been reported to be reduced in both the BAT and WAT of obese animal models (Ruderman et al., 2010). Consistent with these reports, we also demonstrated that mice with adipocyte AMPKα ablation were predisposed to HFD-induced obesity. To assess the anti-obesity efficacy of chronic AMPK activation *in vivo*, we investigated the effect of the administration of A-769662, an AMPK allosteric activator, in chow- and HFD-fed mice. A-769662 (30 mg/kg/day) was administered q.d. for 6 weeks. During treatment, A-769662 did not affect food intake in either chow-fed or HFD-fed mice (Figure 6B). In chow-fed mice, body weight gain was generally unchanged by A-769662 treatment. Meanwhile, in HFD-fed mice, body weight gain was significantly decreased (by 49%) during the 6 weeks of A-769662 treatment (Figure 6A). A-769662 did not alter WAT or liver weight in chow-fed mice, but in HFD-fed mice, the weight of the liver, iWAT and pWAT was reduced by 15, 23, and 13%, respectively, but the eWAT weight was unchanged (Figure 6C). Accordingly, the content of TG and TC in liver were significantly decreased by A-769662 treatment in HFD-fed mice (Figures 6D,E). The fasting blood glucose level was not affected by A-769662 treatment in chow-fed mice or HFD-fed mice (Figure 6F). Nevertheless, A-769662 improved glucose tolerance in HFD-fed mice, as evidenced by the approximately 12% decrease in the AUC (Figures 6G,H), while had no effect on chow-fed mice. Insulin tolerance test showed that A-769662 treatment did not improve insulin sensitivity in HFD-fed mice (Figures 6I,J). In chow-fed mice, the plasma parameters were not significantly changed by A-769662 treatment (Table 2). However, in HFD-fed mice, the plasma levels of TG, TC and low-density lipoprotein cholesterol (LDL-C) were decreased by 27, 36, and 27% (Table 2), respectively. In addition, the insulin and leptin levels were also decreased by 41 and 56% (Table 2), respectively. Together, these results revealed that A-769662 had anti-obesity and anti-hyperlipidemic effects, and this agonist improved glucose tolerance in HFD-fed mice but not in chow-fed mice, indicating that chronic AMPK activation might be specifically effective in the obese mouse model and have negligible adverse effects in normal lean mice.

**Table 2:** Chronic effects of A-769662 on plasma metabolic variables in chow-fed mice and HFD-fed mice.

**Parameters**	**Chow-Veh**	**Chow-A-769662**	**HFD-Veh**	**HFD-A-769662**
TC (mM)	3.58 ± 0.37	3.64 ± 0.47	8.04 ± 0.52	5.12 ± 0.56^***^
LDL-C (mM)	0.66 ± 0.08	0.57 ± 0.03	1.66 ± 0.11	1.21 ± 0.16^*^
HDL-C (mM)	0.44 ± 0.04	0.45 ± 0.07	0.58 ± 0.04	0.54 ± 0.07
LDL-C/HDL-C	1.47 ± 0.08	1.29 ± 0.24	3.09 ± 0.37	2.70 ± 0.45
TG (mM)	0.50 ± 0.04	0.53 ± 0.06	0.46 ± 0.02	0.34 ± 0.03^**^
NEFA (mEq/L)	0.62 ± 0.06	0.64 ± 0.09	0.52 ± 0.05	0.45 ± 0.03
Insulin (ng/ml)	1.51 ± 0.13	1.67 ± 0.04	4.64 ± 0.79	2.74 ± 0.32^*^
Leptin (ng/ml)	1.99 ± 0.56	2.10 ± 0.33	68.42 ± 7.07	30.31 ± 5.05^***^
Irisin (ng/ml)	108.85 ± 6.21	102.52 ± 4.06	109.04 ± 3.28	106.59 ± 4.40

*Values are expressed as the means ± SEM. n = 6–10. Student's t-test*.

* *p < 0.05*,

** *p < 0.01*,

*** *p < 0.001 compared with the corresponding vehicle group*.

### A-769662 promoted energy expenditure in HFD-fed mice

**Figure 7:**
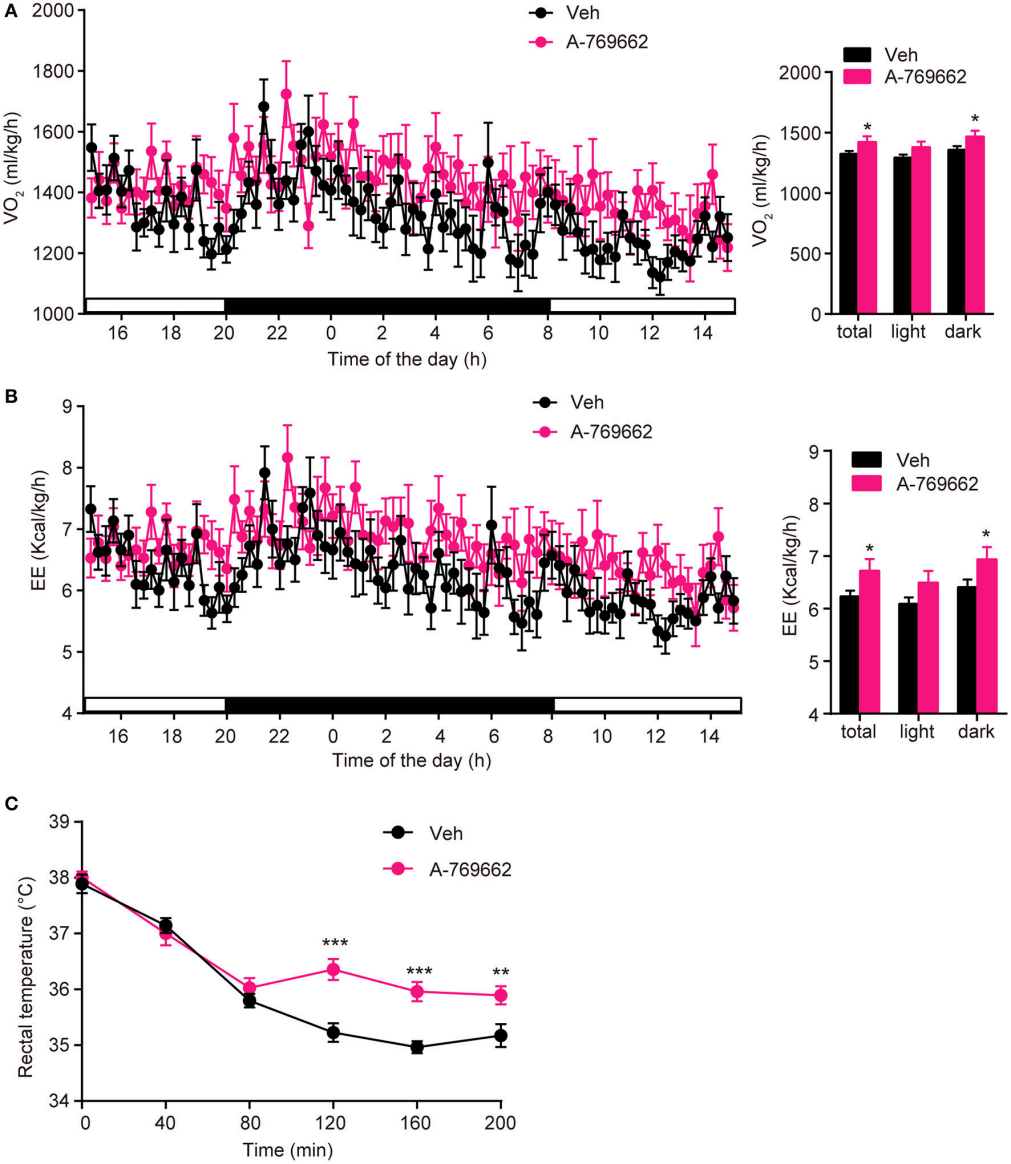
A-769662 promoted energy expenditure and cold tolerance in HFD-fed mice. **(A,B)** During the 4th week of chronic administration, indirect calorimetry was used to investigate the energy metabolism of HFD-fed mice. Mice were dosed with vehicle or A-769662 (30 mg/kg) and then acclimated to the chamber for 12 h. Change of O_2_ consumption (**A** left), EE (**B** left) during the indicated periods and average O_2_ consumption (**A** right), EE (**B** right) during the whole period of measurement were assessed. *n* = 8. **(C)** Change in body temperature of HFD-fed mice after cold exposure at 4°C. *n* = 11–12. Data are presented as the means ± SEM. Student's *t*-test. ^*^P < 0.05, ^**^P < 0.01, and ^***^P < 0.001 compared with the indicated control group.

After observing the anti-obesity effects of A-769662 on HFD-fed mice, we further investigated the effect of A-769662 treatment on whole-body energy expenditure of HFD-fed mice at 4th week of treatment by using indirect calorimetry. The last administration was given 4 h before the experiment. The animals were monitored for O_2_ consumption, EE and locomotor activity for 24 h. A-769662 increased the total and dark-phase O_2_ consumption and EE in HFD-fed mice (Figures 7A,B), but did not induce any significant changes in RER or locomotor activity (Supplementary Figures 5A,B). Additionally, A-769662-treated group exhibited a higher body temperature during the 120–200 min after cold exposure at 4°C (Figure 7C), suggesting that A-769662 protected against hypothermia via enhanced adaptive thermogenesis. Meanwhile, the O_2_ consumption and EE of chow-fed mice were unchanged by A-769662 treatment (data not shown). These results suggest that the anti-obesity effects of A-769662 on HFD-fed mice might be due to increased energy expenditure.

### A-769662 induced browning signature in the iWAT of HFD-fed mice

**Figure 8:**
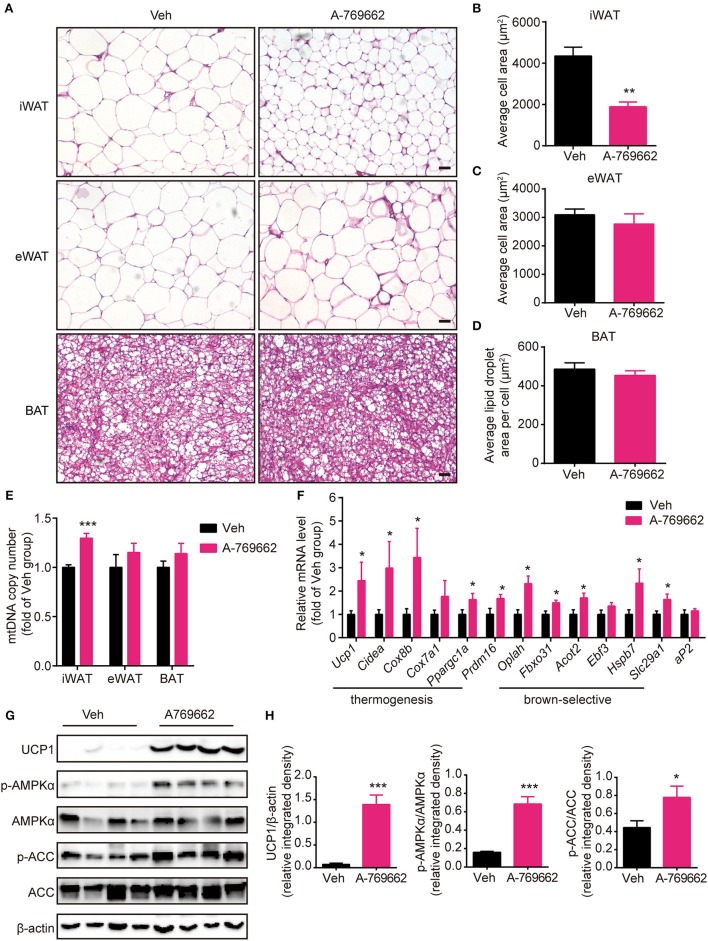
A-769662 induced browning in the inguinal WAT of HFD-fed mice. **(A)** Representative H&E-stained images of the iWAT, eWAT and BAT of HFD-fed mice. Scale bar = 100 μm. **(B–D)** Average adipocyte area in the iWAT **(B)** and eWAT **(C)** and average lipid droplet area in the BAT **(D)** of HFD-fed mice. *n* = 4–6. **(E)** Mitochondrial DNA copy number of iWAT, eWAT, and BAT in HFD-fed mice. **(F)** Relative mRNA levels of thermogenic genes and brown-selective genes in the iWAT of HFD-fed mice after 6 weeks of treatment. *n* = 6–8. **(G)** Western blot analysis of UCP1, AMPKα, p-AMPKα (T172), ACC, and p-ACC (S79) expression levels in the iWAT of HFD-fed mice after 6 weeks of treatment. β-actin was used as a loading control. **(H)** Relative protein expression level of UCP1 and the relative phosphorylation levels of AMPKα and ACC were determined by densitometric quantification of the immunoblots shown in **(G)**. *n* = 4. Data are presented as the means ± SEM. Student's *t*-test. ^*^P < 0.05, ^**^P < 0.01, ^***^P < 0.001 compared with the indicated control group.

Brown and beige fat specialize in energy expenditure through thermogenesis (Harms and Seale, 2013). Since we observed an increase in EE and resistance to cold exposure following A-769662 treatment in HFD-fed mice, we further investigated the cellular and molecular mechanism of A-769662-induced thermogenesis *in vivo*. The weight (Figure 6C) and histological morphology (Figures 8A,D) of BAT and the expression of thermogenesis-related genes in the BAT (Supplementary Figure 6A) were all unchanged by A-769662 treatment. In accordance with the reduced iWAT weight induced by A-769662 treatment, the adipocyte area in iWAT was reduced by approximately 57% (Figures 8A,B). And mitochondria content in iWAT, but not in eWAT or BAT, was elevated by A-769662 treatment (Figure 8E), suggesting that AMPK activation enhanced mitochondrial biogenesis in iWAT. Moreover, the mRNA levels of the BAT-specific marker *Ucp1* and other thermogenesis-related genes, including *Cidea, Cox8b, Cox7a1*, and *Ppargc1a*, in iWAT were significantly up-regulated by A-769662 treatment, and most of the brown-selective markers, such as *Oplah, Fbxo31, Acot2, Hspb7*, and *Slc29a1* (Wu et al., 2012), were also increased (Figure 8F), indicating a genetic conversion of iWAT into beige adipose induced by A-769662. We also detected the expression of the general adipogenic marker *aP2*, and it was unaffected by treatment (Figure 8F), implying that A-769662 may have no impact on the differentiation of inguinal adipocytes. However, the adipocyte area and expression of thermogenesis-related genes in the eWAT were unaltered (Figures 8A,C, Supplementary Figure 6B). After observing an increase in the expression of thermogenic genes in iWAT, we further investigated the expression levels of thermogenic proteins. We found that the protein levels of UCP-1 and PGC-1α were markedly increased in iWAT (Figures 8G,H). Simultaneously, AMPK signaling in iWAT was activated by A-769662 treatment, as evidenced by the increased phosphorylation of AMPKα and its downstream substrate ACC (Figures 8G,H), which suggests that chronic AMPK activation by A-769662 induced iWAT browning in diet-induced obese mice. Taken together, these results indicate that A-769662 specifically promote browning in the iWAT of HFD-fed mice.

### A-769662 facilitated thermogenesis *in vitro* through AMPK signaling pathway

**Figure 9:**
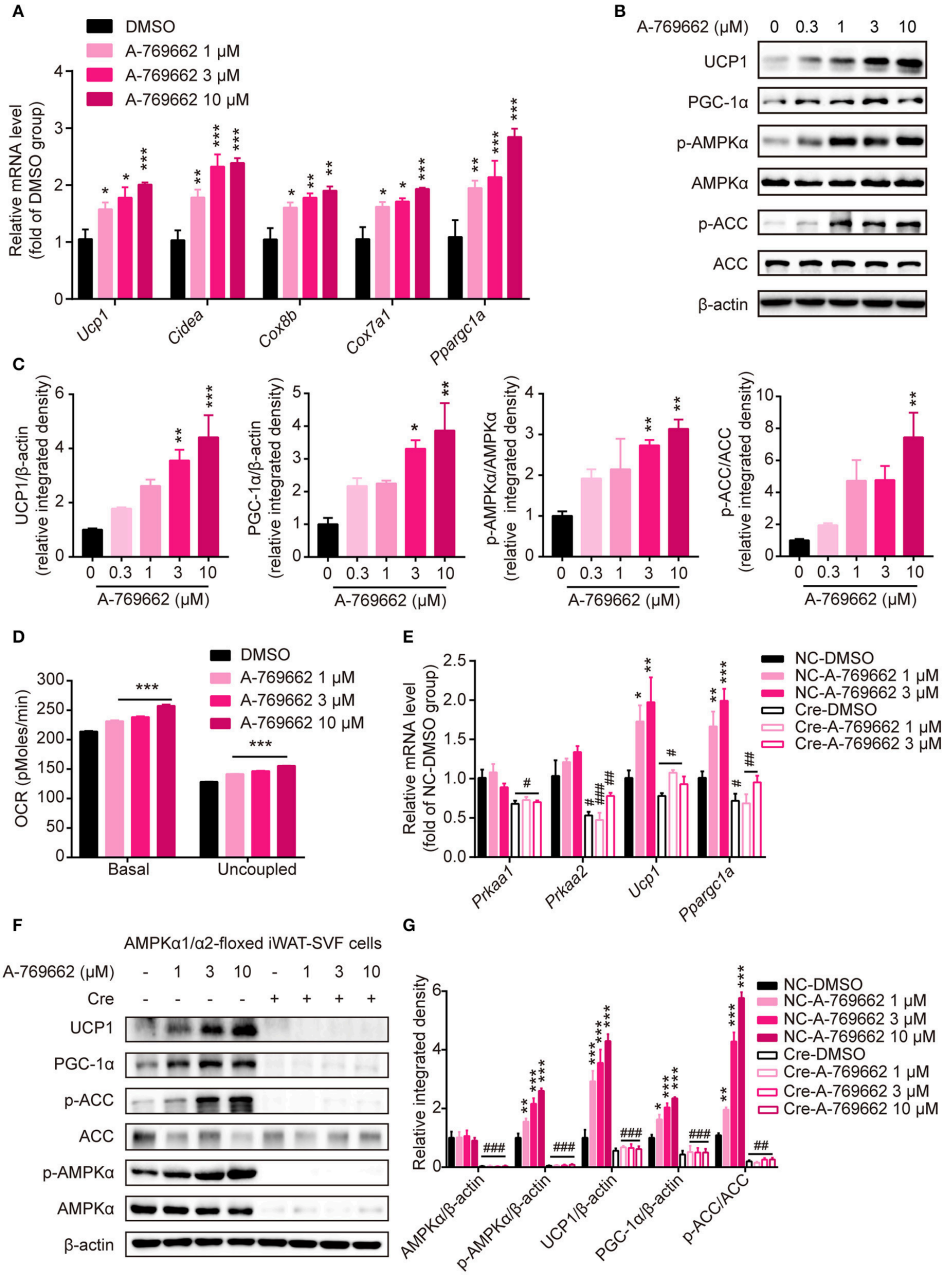
A-769662 facilitated thermogenesis in differentiated iWAT-SVF cells through AMPK signaling pathway. **(A–D)** iWAT-SVF cells were induced to differentiation toward brown-like adipocytes and were treated with the indicated compounds on day 7. **(A)** Relative mRNA levels of thermogenic genes in differentiated iWAT-SVF cells treated with the indicated compounds for 6 h were analyzed by quantitative RT-PCR. *n* = 4. **(B)** Western blot analysis of UCP1, PGC-1α, p-AMPKα (T172), AMPKα, p-ACC (S79), and ACC expression levels in differentiated iWAT-SVF cells treated with indicated compounds on day 7 for 24 h. **(C)** Relative protein expression levels of UCP1 and PGC-1α and the relative phosphorylation levels of AMPKα and ACC were determined by densitometric quantification of the immunoblots shown in (**B**). n = 3. **(D)** Basal and uncoupled oxygen consumption rate (OCR) of differentiated iWAT-SVF cells treated with NE (10 μM) for 3 h or with DMSO or A-769662 at different concentrations on day 7 for 12 h. *n* = 4. **(E–G)** iWAT-SVF cells were isolated from iWAT of 5-week-old AMPK α1/α2-floxed mice and induced to differentiate toward beige adipocytes. Cells were infected with NC and Cre lentivirus on day 6 to knockdown AMPKα expression and were treated with the indicated compounds on day 8. **(E)** Relative mRNA levels of the indicated genes in differentiated iWAT-SVF cells treated with the indicated compounds for 6 h on day 8 were analyzed by quantitative RT-PCR. *n* = 4. **(F)** Western blot analysis of UCP1, PGC-1α, p-AMPKα (T172), AMPKα, p-ACC (S79), and ACC expression levels in differentiated iWAT-SVF cells treated with indicated compounds on day 8 for 24 h. **(G)** Relative protein expression levels of UCP1 and PGC-1α and the relative phosphorylation levels of AMPKα and ACC were determined by densitometric quantification of the immunoblots shown in **(F)**. *n* = 3. Data are presented as the means ± SEM. One-way ANOVA. ^*^P < 0.05, ^**^P < 0.01, ^***^P < 0.001 compared with the indicated DMSO group; ^#^*P* < 0.05, ^##^*P* < 0.01, ^###^*P* < 0.001 compared with the indicated NC group.

To explore the effect of A-769662-induced AMPK activation on differentiated adipocytes in a cell-autonomous manner, SVF cells from iWAT were induced to differentiate into beige adipocytes and were treated with DMSO or A-769662 at the indicated concentration on day 7. The expression of thermogenesis-related genes, such as *Ucp1, Cidea, Cox8b, Cox7a1*, and *Ppargc1a*, was significantly increased by A-769662 treatment in a concentration-dependent manner (Figure 9A). Importantly, similar to the *in vivo* results, the expression of thermogenic protein UCP1 and of PGC-1α was also remarkably increased in differentiated iWAT-SVF cells, which was concomitant with the activation of AMPK signaling by A-769662 treatment (Figures 9B,C). In addition, the basal and uncoupled O_2_ consumption rates (OCRs) were both up-regulated by A-769662 treatment in differentiated iWAT-SVF cells (Figure 9D). To confirm whether the effect of A-769662 on promoting thermogenesis was dependent on AMPK activation, primary SVF cells isolated from iWAT of the floxed mice were induced to differentiate into beige adipocytes, followed by adding Cre lentivirus at day 6 of differentiation to knockdown AMPKα expression, and were then treated with A-769662 at indicated concentration on day 8. As a result, the expression of genes encoding AMPKα1 and AMPKα2 (*Prkaa1* and *Prkaa2*, respectively) and total AMPKα in iWAT-SVF cells were significantly reduced and the activation of AMPK signaling by A-769662 was successfully blocked after Cre lentivirus infection (Figures 9E–G). Importantly, A-769662 treatment up-regulated the expression of thermogenesis marker *Ucp1* and *Ppargc1a* and their encoding products, which were remarkably blunted after AMPKα knockdown (Figures 9E–G), suggesting that AMPK activation was required for A-769662-facilitated thermogenesis. These data indicate that A-769662 promotes thermogenesis *in vitro* via AMPK signaling pathway, which may contribute to A-769662-induced browning in iWAT *in vivo*.

## Discussion

Since obesity has become a global health problem and efforts to reduce energy intake show either limited effectiveness or unacceptable side effects (James et al., 2010), therapeutic strategies aimed at increasing EE are an attractive approach for combatting obesity (Tam et al., 2012). Approaches attempting to enhance the thermogenic activity of brown and beige fat may be beneficial for obesity therapy because the activity of brown and beige fat have been shown to be inversely correlated with BMI and fat mass and positively related to EE in humans (Saito et al., 2009; van Marken Lichtenbelt et al., 2009). Reduced adipose tissue AMPK activity is generally observed in many obese and diabetic animal models as well as in obese humans with insulin resistance (Yu et al., 2004; Ruderman et al., 2010; Gauthier et al., 2011; Xu et al., 2012). The physiological relevance of lowered AMPK activity in adipose tissue to whole-body adiposity remains ambiguous. Here, we found that ablation of adipocyte AMPKα impaired adaptive thermogenesis and EE in response to cold exposure or β3-adrenergic stimulation, and predisposed HFD-fed mice to obesity, glucose intolerance and insulin resistance. Importantly, the lack of adipocyte AMPKα selectively blunted cold-induced thermogenic protein expression in iWAT, resulting in reduced energy utilization and increased adipocyte size, indicating that adipocyte AMPKα is required for browning in iWAT. In contrast, A-769662-induced direct AMPK activation promoted browning in iWAT, which may contribute to the reduced adiposity and improved glucose and lipid metabolism observed in A-769662-treated HFD-fed mice. In summary, these findings indicate that reduced AMPK activity in adipose tissue might be an important pathogenic factor in obesity and the related metabolic syndrome, and the chronic activation of AMPK by A-769662 protects from HFD-induced adiposity and metabolic dysfunction.

Since both catalytic subunits AMPK α1 and α2 exist in adipose tissue and the deletion of a single subunit often leads to the up-regulated activity of the other subunit (Bauwens et al., 2011), we generated AKO mice by crossing Adiponectin-Cre mice with AMPK α1/α2-foxed mice in order to investigate the metabolic role of adipocyte AMPKα. The Adiponectin-driven Cre recombinase is supposed to be expressed at the beginning during the early stage of BAT development (Cohen et al., 2014). When fed a chow diet, AKO mice started to gain more weight than age-matched floxed littermates at 29 weeks of age, however, when fed a HFD, this difference occurred, at 25 weeks of age, indicating that the loss of adipocyte AMPKα accelerates the development of obesity in response to a HFD. These phenotypes were analogous to the inducible mouse model with adipocyte AMPK β1/β2 deletion (iβ1β2AKO) after 8 weeks of age previously reported by Mottillo et al. (2016). The iβ1β2AKO mice had defects not only in the browning of WAT but also in BAT-mediated thermogenesis due to impaired mitochondrial integrity and function but not mitochondrial biogenesis. Intriguingly, we observed that there were not only defects in mitochondrial structure but also reduced total number of mitochondria in iWAT and BAT of AKO mice. However, the thermogenic capacity of BAT from the AKO mice in our study was normal compared with that of the floxed mice, suggesting that AMPK might play different roles in BAT function at different ages and that the unaffected thermogenesis of BAT in AKO mice might be remedied through an unknown mechanism. In addition, both hepatic steatosis and fibrosis were developed in HFD-fed AKO mice, which is more severe than that in iβ1β2AKO mice. This may be attributed to AMPK deletion at earlier stage and longer period of HFD challenge in AKO mice compared to that of iβ1β2AKO mice (Mottillo et al., 2016). It is well-established that adipose tissue-liver crosstalk plays a vital role in regulating systemic glucose and lipid metabolism (Stern et al., 2016). Adiponectin ameliorates liver ectopic lipid accumulation by inhibiting hepatic lipogenesis and enhancing β-oxidation. The plasma level of adiponectin was reduced in chow- and HFD-fed AKO mice, which may intensify the development of hepatic steatosis and even fibrosis. Accumulative evidence have shown that leptin plays a crucial role in the development of liver fibrosis (Marra, 2002). The plasma level of leptin increased along with increased fat mass in AKO mice, which partially explains the aggravating progression of liver fibrosis in AKO mice. Besides, another study reported by Sun-Joong *et al*. showed that adipose tissue-specific AMPK α1/α2 KO mice using Adiponectin-Cre or aP2-Cre model displayed a lean phenotype due to enhanced lipolysis in adipose tissue (Kim et al., 2016). The Adiponectin-Cre model is known to have better efficiency and specificity for adipocytes than the aP2-Cre model (Jeffery et al., 2014), so Adiponectin-Cre model is more suitable for the study of adipose tissue. After comparing the phenotypes of the adiponectin-driven AMPK α1/α2 KO mice used in our study and that by Sun-Joong et al., several difference were found. The adiponectin-driven AMPK α1/α2 KO mice in their study started to gain less body weight and fat mass at 10 weeks of age, while the body weights of our mice showed no significant change between genotypes, and the fat mass was not determined at that time point (Figure 2A). In addition, the body weight change in adiponectin-driven AMPK α1/α2 KO mice after 12 weeks of age was not shown, and the long-term effects of adipocyte AMPK deletion on whole-body metabolism remained unknown in their study. However, despite unaltered body weight, defective adaptive thermogenesis and cold tolerance were observed in the AKO mice in our study at 8 weeks of age, which may lead to reduced EE and accumulated adiposity during aging and in response to the HFD challenge. One possible reason for the differences in the phenotypes of their study and ours might be that the mice used in the experiments were at different ages.

AMPK signaling is indirectly activated by catecholamine secretion in response to cold or β-adrenergic stimulation (Gauthier et al., 2008). We also showed that the sensitivity of AMPK activation and UCP1 expression in different fat depots were diverse, with iWAT being the most responsive to cold exposure, and eWAT and BAT being less responsive to the cold stimulation (Figure 1A and Supplementary Figure 1A). Ablation of adipocyte AMPKα specially impaired mitochondria quality control and biogenesis in iWAT and BAT but not in eWAT (Figures 1J–M and Supplementary Figure 1M). Accordingly, the adipocyte size in the iWAT and BAT of AKO mice was increased while that in eWAT were unchanged (Figures 3A–D). Nonetheless, the expression of the thermogenesis-related protein was markedly reduced in iWAT but not in BAT of AKO mice. In addition, chronic AMPK activation by A-769662 treatment selectively promoted browning in the iWAT of HFD-fed mice but not in eWAT or BAT (Figures 8A–H and Supplementary Figures 6A,B). Our observations are in line with previous reports that eWAT is more “resistant” to browning than iWAT fat depots, while iWAT has greater plasticity and ability to modulate metabolic function upon stimulation, as evidenced by the up-regulation of brown-selective marker genes in response to cold or β-adrenergic stimulation (Bartelt and Heeren, 2014). Despite the unique structural and molecular characteristics of different fat depots that cause these depot-specific differences, our findings suggest that the high sensitivity of AMPK activation in iWAT may contribute to its plasticity in adaptation to various environmental and hormonal cues.

A previous study reported that the improvement in blood glucose level from A-769662 treatment was mainly due to targeting liver tissue, and A-769662 treatment reduced food intake in a 14-day study with *ob/ob* mice (Fullerton et al., 2013). In this study, we also observed that A-769662 ameliorated glucose and lipid disorders in HFD-fed mice (Figure 6 and Table 2). However, in our model, food consumption was unchanged during the 6 weeks of study, ruling out the disturbance of energy intake differences in the anti-obesity effect of A-769662. Besides liver, WAT is another target tissue of A-769662, as evidenced by the tissue weight, morphology, related gene expression and UCP-1 protein level. Moreover, there was a considerable amount of A-769662 distribution in the adipose tissue of HFD-fed mice (Supplementary Figure 7), suggesting a direct effect of A-769662 on adipose tissue. In this study, we observed that A-769662 treatment augmented whole-body EE and enhanced adaptive thermogenesis in HFD-fed mice, and these effects were not driven by increased locomotor activity (Figures 7A,B and Supplementary Figure 5B). BAT activity plays a primary role in cold-induced adaptive thermogenesis and EE in rodents (Tam et al., 2012). However, in our study, the mRNA levels of thermogenesis-related genes were not up-regulated by A-769662 in the BAT (Supplementary Figure 6A). These results seem to rule out the possibility that BAT plays a predominant role in the A-769662-induced enhancement of EE and adaptive thermogenesis. Skeletal muscle, as a large organ and major site of facultative thermogenesis, also contributes to EE (Zurlo et al., 1990), but the gene expression of *Ppargc1a*, an AMPK downstream substrate and key regulator of mitochondrial biogenesis and oxidative phosphorylation (OXPHOS) (Arany, 2008), was not changed by A-769662 in the skeletal muscle of HFD-fed mice (Supplementary Figure 8). Meanwhile, the mRNA levels of *Ppargc1a* and other thermogenic genes, and the protein level of UCP1 were increased by A-769662 in iWAT; in contrast, the expression levels of cold-induced UCP1 and PGC-1α protein were remarkably reduced in iWAT with adipocyte AMPK deletion. Therefore, it is possible that the A-769662-induced improvement of metabolic disorders in obese mice is at least partly due to increased EE through the browning of iWAT.

There are also studies suggesting that long-term AICAR treatment promotes energy dissipation in chow-fed rat models (Gaidhu et al., 2009, 2011) and improves glucose homeostasis and insulin resistance in diabetic mouse models (Buhl et al., 2002; Song et al., 2002). AICAR treatment also had an inhibitory effect on food consumption in these chronic studies. In our study with long-term A-769662 treatment, the chow-fed mice exhibited no effect on food intake, EE or fat metabolism, which may be due to variations in species, treatment duration, dosage and injection frequency in different animal models. Actually, it was reported that no detectable distribution of A-769662 into the brain of *ob/ob* mice (1 h after a single injection at a dose of 30 mg/kg; Cool et al., 2006), which suggests that the absence of effect of A-769662 on food intake may be owing to no exposure of A-769662 into the brain. Although AICAR and A-769662 are both AMPK activators, they have different mechanisms. AICAR activates AMPK by being taken up into cells and converted into an AMP mimetic, ZMP (Hardie, 2011). Similar to AMP, A-769662 allosterically activates AMPK and inhibits the dephosphorylation of AMPK (Thr-172). However, AMPK activation by A-769662 is exclusively dependent on the existence of glycogen binding domain within the β1 subunit, but not the γ subunit to which AMP binding (Sanders et al., 2007). The absence of effect on chow-fed mice is also consistent with the study conducted by Cool et al. (2006). In our study, the pro-browning effect of A-769662 only existed in the HFD-fed mice, and this may be owing to differences in metabolism and energy status of HFD-fed and chow-fed mice. This result suggests that in mouse models, the anti-obesity effect of chronic AMPK activation is more sensitive and responsive in diet-induced obese mice.

Beige adipocytes have been reported to arise from two alternative processes: *de novo* differentiation from progenitor cells and trans-differentiation from white adipocytes (Bartelt and Heeren, 2014). In general, the differentiation of brown adipocytes shares most of the transcriptional regulation pathways with beige adipocytes (Inagaki et al., 2016). Some studies have reported that AMPK plays a positive role in brown adipocyte differentiation and brown fat development in murine cell lines and animal models (Vila-Bedmar et al., 2010). AMPK activation by AICAR treatment in human adipose-derived mesenchymal stem cells (hADMSCs) from pericardial adipose tissue is reported to induce a morphological change similar to beige adipocytes, but without an actual change of metabolic function (Abdul-Rahman et al., 2016). The restricted effect of AICAR on inducing beige adipogenesis might due to the differences in species or original depots of cells and basal activity/expression level of AMPK or the intrinsic specificity of AICAR. However, the indirect activation of AMPK by berberine has been demonstrated to promote WAT browning by enhancing thermogenesis in mature beige adipocytes via the AMPK-PGC-1α pathway (Zhang et al., 2014). Similarly, direct AMPK activation by A-769662 did not induce the differentiation of iWAT-SVF cells toward beige adipocytes *in vitro* (data not shown) and had no effect on the expression of the adipogenic marker *aP2* in iWAT *in vivo* (Figure 8F). Alternatively, A-769662 enhanced thermogenesis in differentiated iWAT-SVF cells, as detected by the increased expression of thermogenic genes and proteins and the up-regulated O_2_ consumption, which is directly reliant on the activation of AMPK signaling (Figures 9A–G). Moreover, we further determined the plasma level of irisin, which has been reported to stimulate the browning of WAT through specific actions on the beige preadipocyte population (Tseng et al., 2008); the plasma irisin level was not affected by A-769662 treatment (Table 2), and the *Fndc5* mRNA level in skeletal muscle was also influenced (Supplementary Figure 8). These results indicate that the browning of WAT in HFD-fed mice by A-769662 is not due to the direct modulation of SVF cell differentiation, but may derive from direct trans-differentiation of mature adipocytes in iWAT.

In summary, our results demonstrated that the ablation of adipocyte AMPKα impairs adaptive thermogenesis and energy expenditure in response to cold and β-adrenergic stimulation, leading to a predisposition for HFD-induced obesity and metabolic dysfunction. Moreover, pharmacological chronic AMPK activation by A-769662 alleviated diet-induced obesity via promoting browning in inguinal WAT. Overall, our findings indicate that AMPK plays a vital role in modulating WAT browning in response to thermal, nutritional and pharmacological cues, supporting chronic AMPK activation as a potentially effective approach for the treatment of obesity and related metabolic diseases through increasing thermogenesis.

## Author contributions

LW, LZ, and BL contributed to study design, data analyzing, discussion and preparation of the manuscript. LW, LZ, BL, HJ, YD, ZX, and LS contributed to conducting the experiments. JYL and JL contributed to study design, discussion, reviewing and editing the manuscript.

### Conflict of interest statement

The authors declare that the research was conducted in the absence of any commercial or financial relationships that could be construed as a potential conflict of interest. The handling Editor declared a shared affiliation, though no other collaboration, with the authors HJ and YD.

## Abbreviations

ACC: acetyl-CoA carboxylase
Acot2: acyl-CoA thioesterase 2
Acta2: smooth muscle actin alpha 2
AICAR: 5-aminoimidazole-4-carboxamide-1-β-D-ribofuranoside
AKT: protein Kinase B
ALT: alanine aminotransferase
AMP: adenosine monophosphate
AMPK: AMP-activated protein kinase
AST: aspartate transaminase
ATP: adenosine triphosphate
BAT: brown adipose tissue
BMI: Body Mass Index
BMP7: bone morphogenetic protein 7
BMP8b: bone morphogenetic protein 8b
Cidea: cell death-inducing DFFA-like effector a
Col1a1: collagen type I alpha 1 chain
Cox5b: cytochrome c oxidase polypeptide 5b
Cox7a1: cytochrome c oxidase polypeptide 7a1
Cox8b: cytochrome c oxidase polypeptide 8b
CPT1: carnitine palmitoyltransferase 1
Ctgf: connective tissue growth factor
Dio2: type II iodothyronine deiodinase
EE: energy expenditure
Elovl3: elongation of very long chain fatty acids protein 3
eWAT: epididymal white adipose tissue
FAO: fatty acid oxidation
Fasn: fatty acid synthase
FBG: fasting blood glucose
Fbxo31: f-box only protein 31
FCCP: carbonyl cyanide 4-(trifluoromethoxy) phenylhydrazone
FGF21: fibroblast growth factor 21
Fndc5: fibronectin type III domain-containing protein 5
Gck: glucose kinase
GTT: glucose tolerance test
hADMSC: human adipose-derived mesenchymal stem cell
HDL-C: high-density lipoprotein cholesterol
H&E: hematoxylin and eosin
HFD: High-fat-diet
Hspb7: heat shock protein family, member 7
IBMX: 3-Isobutyl-1-methylxanthine
IR: insulin receptor
ITT: insulin tolerance test
iWAT: inguinal white adipose tissue
Lcad: long chain acyl-CoA dehydrogenase
LC-MS/MS: liquid chromatography-mass spectrometry/mass spectrometry
LDL-C: low-density lipoprotein cholesterol
Me1: malic enzyme
Mmp2: matrix metallopeptidase 2
mtDNA: mitochondrial DNA
Myf5: myogenic factor 5
NE: norepinephrine
NEFA: nonesterified fatty acid
NMR: 1H-nuclear magnetic resonance
OCR: oxygen consumption rates
Oplah: 5-Oxoprolinase (ATP-Hydrolysing)
OXPHOS: oxidative phosphorylation
PGC-1α: Peroxisome proliferator-activated receptor gamma coactivator 1-alpha
Ppargc1b: Peroxisome proliferator-activated receptor gamma coactivator beta
PRDM16: PR domain containing 16
pWAT: perirenal WAT
RER: respiratory exchange rate
Slc29a1: solute carrier family 29 member 1
SVF: stromal vascular fraction
T3, 3, 3′: 5-Triiodo-L-thyronine
T4: thyroxin
TC: total cholesterol
TG: triglyceride
Timp1: tissue inhibitor of metalloproteinases 1
TRPV4: transient receptor potential cation channel subfamily V member 4
UCP1: uncoupling protein 1
VEGF: vascular endothelial growth factor
WAT: white adipose tissue
ZMP: 5-aminoimidazole-4-carboxamide ribonucleoside monophosphate.
