# Supplementary material for: AMP-Activated Protein Kinase (AMPK) Regulates Energy Metabolism through Modulating Thermogenesis in Adipose Tissue
Source: Front Physiol. 2018 Feb 21;9:122. doi: 10.3389/fphys.2018.00122 (PMC5826329; doi:10.3389/fphys.2018.00122)
Supplement: Supplementary file 1 [file Presentation2.pdf]

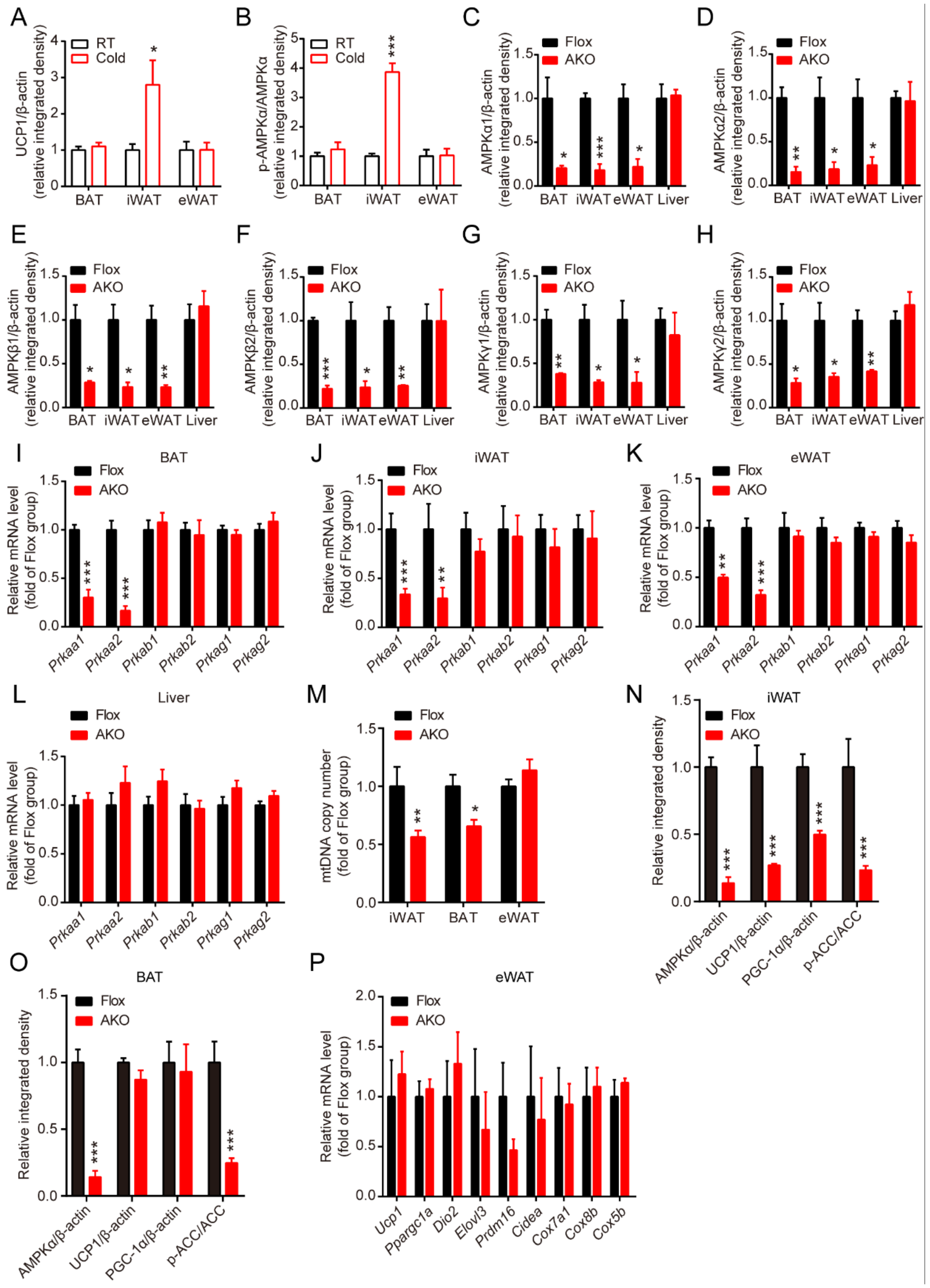

(figure legend on next page)

Supplementary figure 1. Adipose AMPKα was required for cold-induced browning in iWAT. (A-B) Densitometric quantification of immunoblots shown in Figure 1A. n = 4. **(C-H)** Densitometric quantification of immunoblots shown in Figure 1B. n = 3. **(I-L)** The mRNA levels of *Prkaa1*, *Prkaa2*, *Prkab1*, *Prkab2*, *Prkag1* and *Prkag2* in BAT (I), iWAT (J), eWAT (K) (normalized to *36b4*) and liver (L) (normalized to *Actb*) of chow-fed AKO mice and age-matched floxed mice. n = 8-12. **(M)** 8-week-old male chow-fed AKO mice and floxed mice were housed at 4 °C for 48 h. Mitochondrial DNA copy number of iWAT, eWAT and BAT in AKO mice and age-matched floxed littermates were determined. **(N)** Densitometric quantification of immunoblots shown in Figure 1L. n = 3. **(O)** Densitometric quantification of immunoblots shown in Figure 1M. n = 3. **(P)** 8-week-old male chow-fed AKO mice and floxed mice were housed at 4 °C for 48 h. Relative mRNA levels of thermogenic genes in eWAT are shown. n = 9. Data are presented as the means ± SEM. Student's t test. *P < 0.05, **P < 0.01, ***P < 0.001 compared with the indicated control group.

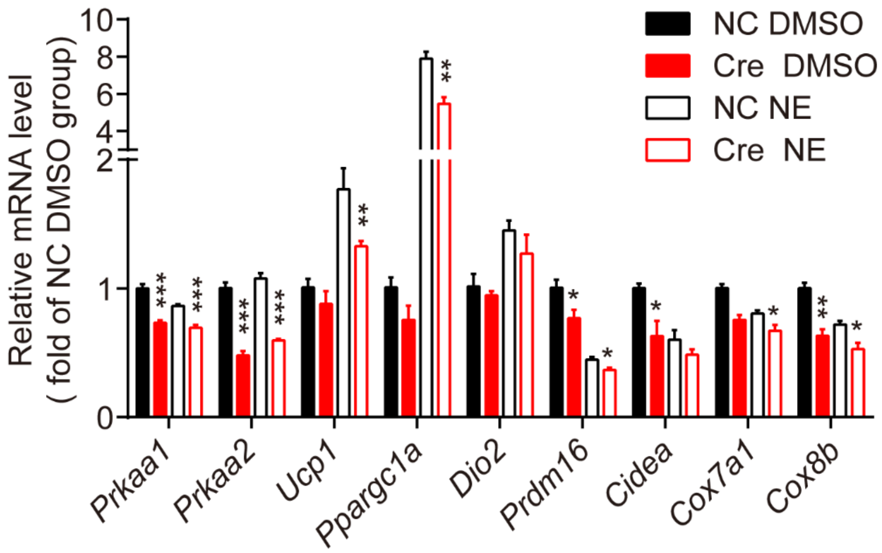

Supplementary figure 2. AMPKα was essential for NE-induced thermogenic genes induction in differentiated iWAT-SVF cells. Inguinal stromal vascular fraction (iWAT-SVF) cells were isolated from iWAT of 5-week-old AMPK α1/α2-floxed mice and induced to differentiate towards beige adipocytes. Cells were infected with NC and Cre adenovirus on day 6 to knockdown AMPKα expression and were treated with NE (10 μM) on day 8 for 6 h. Relative mRNA levels of the indicated genes in iWAT-SVF cells on day 8 were determined by real time Q-PCR. n = 4. Data are presented as the means ± SEM. Student's t test. *P < 0.05, **P < 0.01, ***P < 0.001 compared with the indicated control group.

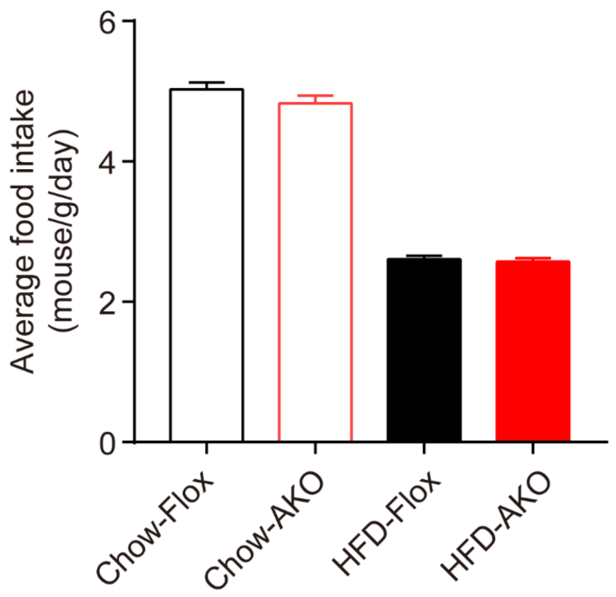

Supplementary figure 3. Deletion of adipocyte AMPKα had no effect on food intake in chow- or HFD-fed mice. Average food intake of AKO mice and age-matched floxed mice fed a chow diet or a HFD during the indicated period. n = 11-15. Data are presented as the means ± SEM.

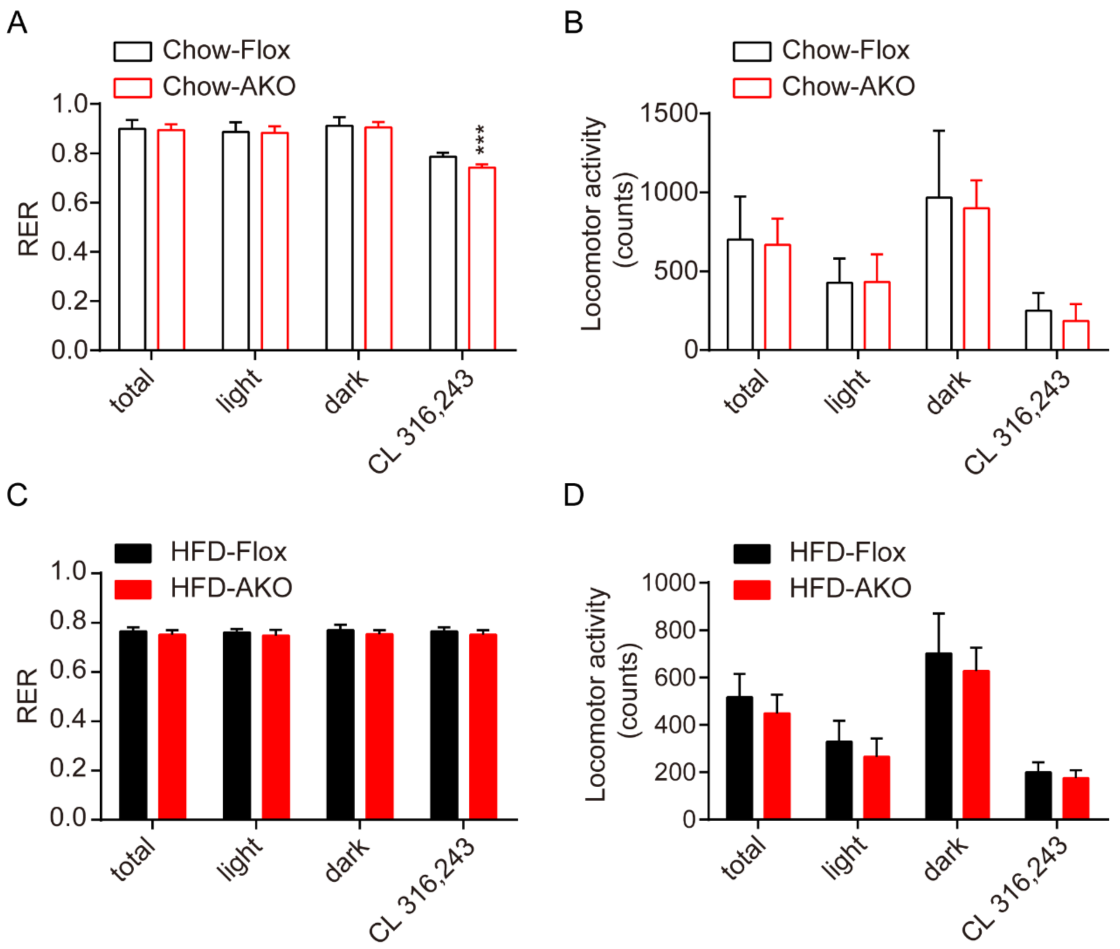

Supplementary figure 4. Effect of adipocyte AMPKα deletion on RER and locomotor activity in AKO mice and floxed mice fed a chow diet or a HFD. (A and C) Average basal and CL 312,643-stimulated RER of AKO mice and floxed mice fed a chow diet (A) or a HFD (C). **(B and D)** Average basal and CL 312,643-stimulated locomotor activity of AKO mice and floxed mice fed a chow diet (B) or a HFD (D). n = 8. Data are presented as the means ± SEM. Student's t test. ***P < 0.001 compared with the indicated control group.

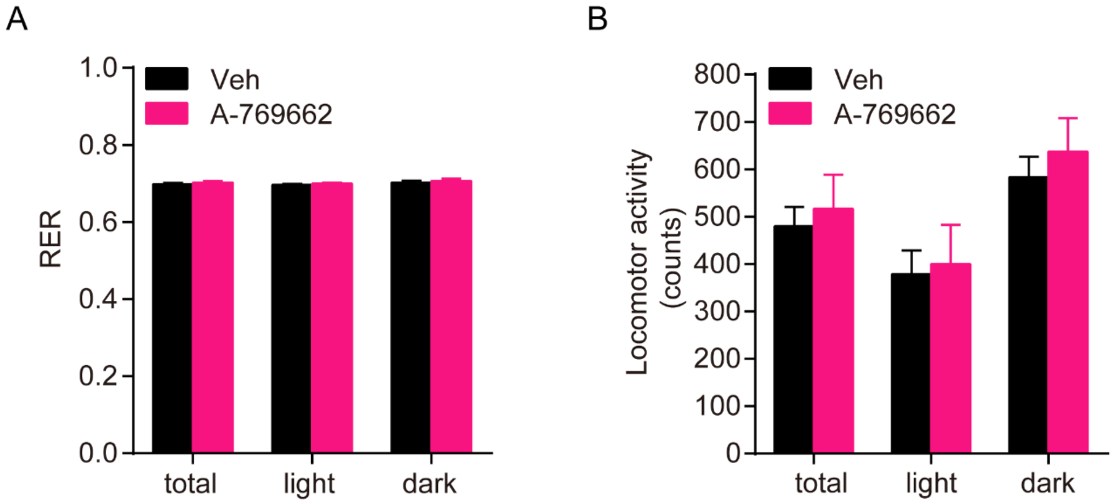

Supplementary figure 5. Chronic A-769662 treatment had no influence on RER and locomotor activity of HFD-fed mice. (A-B) Average RER (A) and locomotor activity (B) of HFD-fed mice was assessed during a 12-h light-dark cycle after 4 weeks of treatment. n = 8. Data are presented as the means ± SEM.

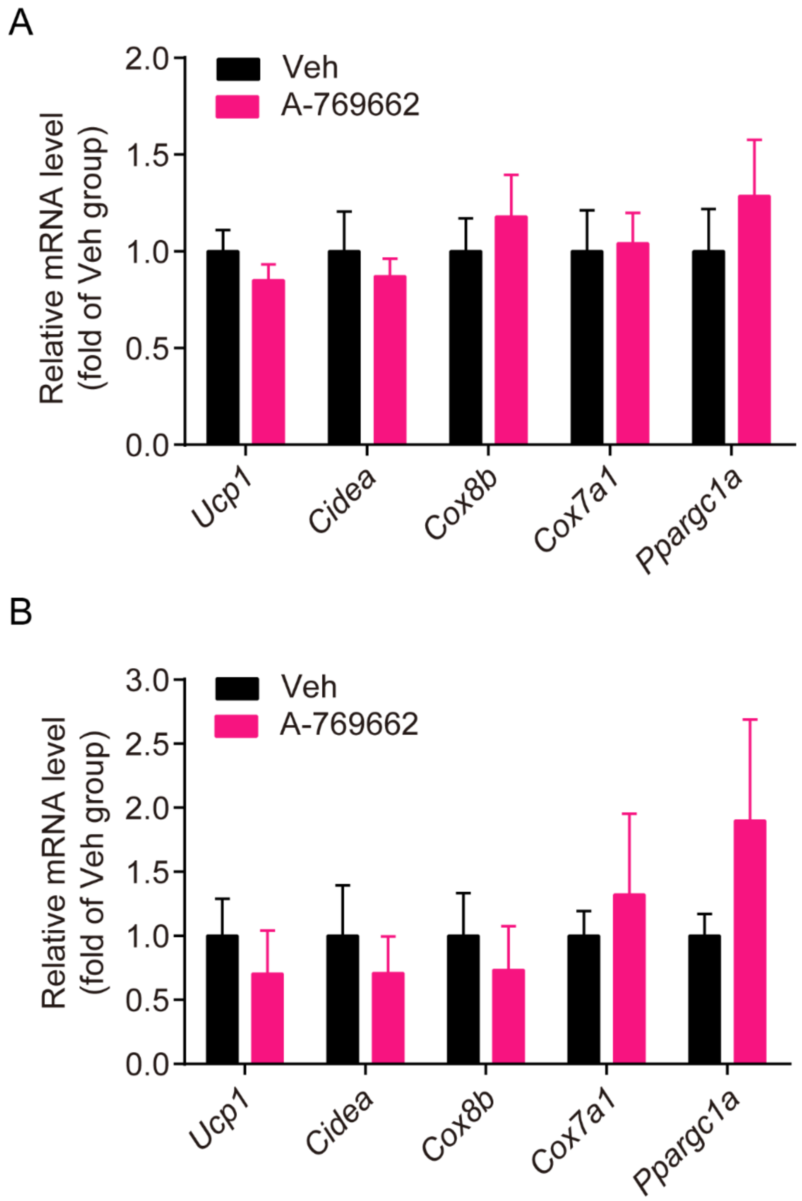

Supplementary figure 6. Chronic A-769662 treatment had no effect on the expression of thermogenic genes in eWAT or BAT of HFD-fed mice. (A-B) Relative mRNA levels of thermogenic genes in BAT (A) and eWAT (B) of HFD-fed mice after 6 weeks of treatment. n = 6-7. Data are presented as the means ± SEM.

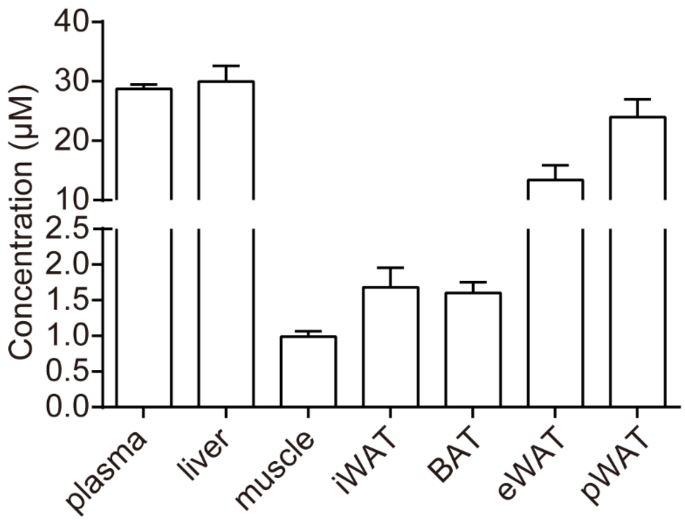

Supplementary figure 7. Tissue distribution of A-769662 in HFD-fed mice. The concentration of A-769662 in different tissues at 1 h after a single dose of i.p. injection of A-769662 (30 mg/kg) in male HFD-fed mice. The plasma and tissue samples were collected and the compound concentrations were determined by LC-MS/MS. n = 3. Data are presented as the means ± SEM.

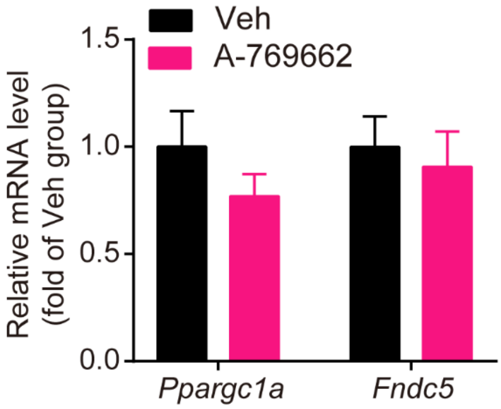

Supplementary figure 8. Effect of A-769662 treatment on gene expression of *Ppargc1a* and *Fndc5* in skeletal muscle of HFD-fed mice. The mRNA levels of *Ppargc1a* and *Fndc5* in skeletal muscle after 6 weeks of treatment (normalized to *Tubulin*). n= 9-10. Data are presented as the means ± SEM.

Supplementary table 1. The sequences of primers used in real time Q-PCR (mouse).

Genes	Sequence
36b4 forward	TTTGGGCATCACCACGAAAA
36b4 reverse	GGACACCCTCCAGAAAGCGA
Tubulin forward	TAGCAGAGATCACCAATGCC
Tubulin reverse	GGCAGCAAGCCATGTATTTA
Actb forward	CACTGTCGAGTCGCGTCC
Actb reverse	TCATCCATGGCGAACTGGTG
Prkaa1 forward	AAAGTGAAGGTGGGCAAGCA
Prkaa1 reverse	CAGATGGTGTACTGATGACCTGG
Prkaa2 forward	TCGCAGTTTAGATGTTGTTGGA
Prkaa2 reverse	CTTCAACCCGCCCATGTTTG
Prkab1 forward	TTCCTTGTGTCCCTGCAGATT
Prkab1 reverse	CCTCTTTCTCTGGAGCCTTGAT
Prkab2 forward	TGGCAGCAGGATTTGGATGAT
Prkab2 reverse	AGGATGGCAACGAAGTCATTATG
Prkag1 forward	AATGAACACTTTCAAGAGACCCC
Prkag1 reverse	CCAACTTGGAACTTGTGGGAAT
Prkag2 forward	GTTGTCTTCGACACTACGTTGC
Prkag2 reverse	ACTCCCTCCACGTTTCAATCTT
Ucp1 forward	ACTGCCACACCTCCAGTCATT
Ucp1 reverse	CTTTGCCTCACTCAGGATTGG
Ppargc1a forward	ACTGAGCTACCCTTGGGATG
Ppargc1a reverse	TAAGAATTTCGGTGGTGACA
Cox8b forward	GAACCATGAAGCCAACGACT
Cox8b reverse	GCGAAGTTCACAGTGGTTCC
Cox7a1 forward	CAGCGTCATGGTCAGTCTGT
Cox7a1 reverse	AGAAAACCGTGTGGCAGAGA
Cox5b forward	GCTGCATCTGTGAAGAGGACAAC
Cox5b reverse	CAGCTTGTAATGGGTTCCACAGT
Ppargc1b forward	GTCCCTGGCTGACATTCACT
Ppargc1b reverse	GCACGGATCTCATGGTCTCT
Prdm16 forward	CAGCACGGTGAAGCCATTC
Prdm16 reverse	GCGTGCATCCGCTTGTG
Cidea forward	TGCTCTTCTGTATCGCCCAGT
Cidea reverse	GCCGTGTTAAGGAATCTGCTG
aP2 forward	ACACCGAGATTTCCTTCAAACTG
aP2 reverse	CCATCTAGGGTTATGATGCTCTTCA
Cpt1b forward	GCACACCAGGCAGTAGCTTT
Cpt1b reverse	CAGGAGTTGATTCCAGACAGGTA
Lcad forward	TCACCAACCGTGAAGCTCGA
Lcad reverse	CCAAAAAGAGGCTAATGCCATG
Oplah forward	CTTCACGCACGTCTCCTTGT
Oplah reverse	GCATCTGCACAGGCCGTAT

Fbxo31 forward	AAACTGCTTCACCGATACAGAC
Fbxo31 reverse	ACCACGACGTTCAGCAATCC
Acot2 forward	ATGGTGGCCTCGTCTTTCG
Acot2 reverse	GAGCGGCGGAGGTACAAAC
Ebf3 forward	CGAAAGGACCGCTTTTGTGG
Ebf3 reverse	AGTGAATGCCGTTGTTGGTTT
Hspb7 forward	GAGCATGTTTTCAGACGACTTTG
Hspb7 reverse	CCGAGGGTCTTGATGTTTCCTT
Slc29a1 forward	CACCAGCCTCAGGACAGGTAT
Slc29a1 reverse	GTCCAGGCGGTTTGTGAAA
Fndc5 forward	ATGAAGGAGATGGGGAGGAA
Fndc5 reverse	GCGGCAGAAGAGAGCTATAACA
Gck forward	TGAGCCGGATGCAGAAGGA
Gck reverse	GCAACATCTTTACACTGGCCT
Me1 forward	GTCGTGCATCTCTCACAGAAG
Me1 reverse	TGAGGGCAGTTGGTTTTATCTTT
Fasn forward	GGAGGTGGTGATAGCCGGTAT
Fasn reverse	TGGGTAATCCATAGAGCCCAG
Acc1 forward	TCTACGGCAGCAGTTACACCACAT
Acc1 reverse	TCTCTTCATTACCTCAATCTCAGCATAG
Scd1 forward	CAGGTTTCCAAGCGCAGTTC
Scd1 reverse	ACTGGAGATCTCTTGGAGCA
Acta2 forward	TGGCCACTGCTGCTTCCTCTTCTT
Acta2 reverse	GGGGCCAGCTTCGTCATA CTCCT
Col1a1 forward	CAAGGTCCTTCTGGATCAAGTG
Col1a1 reverse	CCTTTATGCCTCTGTCACCTTG
Ctgf forward	CAAGGACCGCACAGCAGTT
Ctgf reverse	AGAACAGGCGCTCCACTCTG
Mmp2 forward	CTGGCATCCTCTTGTTGCTA
Mmp2 reverse	AGGGATCTCCAGGTGCACAA
Timp1 forward	CACGGGCCGCCTAAGGAACG
Timp1 reverse	GGTCATCGGGCCCCAAGGGA
16S rRNA forward	CCGCAAGGGAAAGATGAAAGAC
16S rRNA reverse	TCGTTTGGTTTCGGGGTTTC
Hexokinase 2 gene, intron 9 forward	GCCAGCCTCTCCTGATTTTAGTGT
Hexokinase 2 gene, intron 9 reverse	GGGAACACAAAAGACCTCTTCTGG
